# MRPS6 modulates glucose-stimulated insulin secretion in mouse islet cells through mitochondrial unfolded protein response

**DOI:** 10.1038/s41598-023-43438-7

**Published:** 2023-09-27

**Authors:** Danhong Lin, Jingwen Yu, Leweihua Lin, Qianying Ou, Huibiao Quan

**Affiliations:** Department of Endocrinology, Hainan General Hospital, Hainan Affiliated Hospital of Hainan Medical University, No.19 Xiuhua Road, Haikou, 570311 Hainan China

**Keywords:** Endocrine system and metabolic diseases, Type 2 diabetes, Gene regulation, Mitochondria

## Abstract

Lack of efficient insulin secretion from the pancreas can lead to impaired glucose tolerance (IGT), prediabetes, and diabetes. We have previously identified two IGT-associated single nucleotide polymorphisms (SNPs) rs62212118 and rs13052524 located at two overlapping genes: MRPS6 and SLC5A3. In this study, we show that MRPS6 but not SLC5A3 regulates glucose-stimulated insulin secretion (GSIS) in primary human β-cell and a mouse pancreatic insulinoma β-cell line. Data mining and biochemical studies reveal that MRPS6 is positively regulated by the mitochondrial unfolded protein response (UPR^mt^), but feedback inhibits UPR^mt^. Disruption of such feedback by MRPS6 knockdown causes UPR^mt^ hyperactivation in high glucose conditions, hence elevated ROS levels, increased apoptosis, and impaired GSIS. Conversely, MRPS6 overexpression reduces UPR^mt^, mitigates high glucose-induced ROS levels and apoptosis, and enhances GSIS in an ATF5-dependent manner. Consistently, UPR^mt^ up-regulation or down-regulation by modulating ATF5 expression is sufficient to decrease or increase GSIS. The negative role of UPR^mt^ in GSIS is further supported by analysis of public transcriptomic data from murine islets. In all, our studies identify MRPS6 and UPR^mt^ as novel modulators of GSIS and apoptosis in β-cells, contributing to our understanding of the molecular and cellular mechanisms of IGT, prediabetes, and diabetes.

## Introduction

Diabetes is a global public health concern^1^. High blood glucose levels in diabetes patients can cause cardiovascular disease, kidney disease, nerve damage, and other complications^2^. Insulin is a hormone that plays a critical role in regulating blood glucose levels^3^. In response to increased blood glucose levels, insulin is released from the beta cells of the pancreas into circulation and binds to insulin receptors in target tissues (liver, muscle, and adipose tissues). Once bound to insulin, the receptors signal the target tissues to uptake glucose into the circulation, therefore keeping blood glucose levels at normal ranges. Malfunctions in insulin secretion from pancreas or glucose uptake in target tissues can lead to prediabetes and diabetes^4^. Different from type 1 diabetes (T1D), where insulin secreting cells are damaged by autoimmunity^5^, T2D can be caused by both reduced insulin secretion by the β-cell and/or reduced glucose uptake by glucose-consuming tissues such as muscles. Impaired glucose tolerance (IGT) is a condition where glucose levels is elevated abnormally, which could further develop into diabetes^4^.

Genome-wide association studies (GWAS) have made significant contributions to our understanding of the genetics of T2D^6,7^. Single nucleotide polymorphisms (SNPs) are common genetic variations in the genome between individuals. GWAS typically involves genotyping hundreds of thousands to millions of SNPs across the genome in large groups of individuals with and without disease or trait of interest^6^. By comparing the frequency of each SNP between the diseased cohorts and normal groups, researchers can identify SNPs that are associated with a disease, providing potential screening makers and clues to understand the disease etiology. GWAS have identified many of genetic variations that are associated with an increased risk of developing T2D^2^. By further genetics and cellular studies, many of these SNP-affected genes are found to be involved in biological processes that are important for glucose metabolism (HNF1A, IRS1, etc.), insulin secretion (TCF7L, 2 KCNJ11, etc.), lipid metabolism, inflammation, and obesity.

Impaired mitochondria functions have been implicated in T2D^8–11^. Mitochondria are the centers for metabolizing glucose and lipid to maintain energy supply through oxidative phosphorylation. In T2D, reduced mitochondrial DNA contents, decreased mitochondrial respiration, and decreased ATP production have been linked to insulin resistance or IGT^12^. One mechanism by which mitochondrial dysfunction contributes to T2D is through the production of reactive oxygen species (ROS). ROS are generated as a byproduct of oxidative phosphorylation and can induce apoptosis in insulin secreting cells and insulin-regulating tissues important for glucose metabolism^13^.

The mitochondrial unfolded protein response (UPR^mt^) is a conserved stress response pathway that is activated in response to protein misfolding and aggregation in the mitochondria^14^. Upon activation, UPR^mt^ upregulates a set of chaperones (such as mtHSP70 encoded by *HSPA9* gene and HSP60 encoded by *HSPD1* gene), proteases (such as LonP1 encoded by LONP1 gene and ClpP encoded by *CLPP* gene), and other proteins that are involved in maintaining protein homeostasis. ATF5 is a major transcription factor required for UPR^mt^ induction of target genes^15^. Interestingly, ATF5 has been shown to regulate β-cell apoptosis in mice^16^. The UPR^mt^ is implicated in a variety of cellular processes and diseases, including aging, cancer, and neurodegeneration^17^. Although mitochondria are well known to affect the development of T2D, whether and how UPR^mt^ would contribute to T2D have not been reported.

By using GWAS study in a cohort of East Asians in China Hainan Province, our previous study has identified two SNPs (rs62212118 and rs13052524) that are associated with 2-h oral glucose tolerance test (2 h-OGTT)^18^, an established indicator of IGT. Both SNPs are located in the intron region of MRPS6 gene, which encodes the mitochondrial ribosome required for protein synthesis inside the mitochondria. In this study, we investigate the molecular and cellular functions of MRPS6 by using genetic manipulation, biochemical studies, cell biology, and data mining. Our studies reveal a novel role of UPR^mt^ in suppressing insulin secretion and promoting apoptosis in mouse pancreatic β-cells, which could help better understand the etiology of IGT, prediabetes, and diabetes.

## Results

### MRPS6 enhanced glucose-stimulated insulin secretion (GSIS)

**Figure 1:**
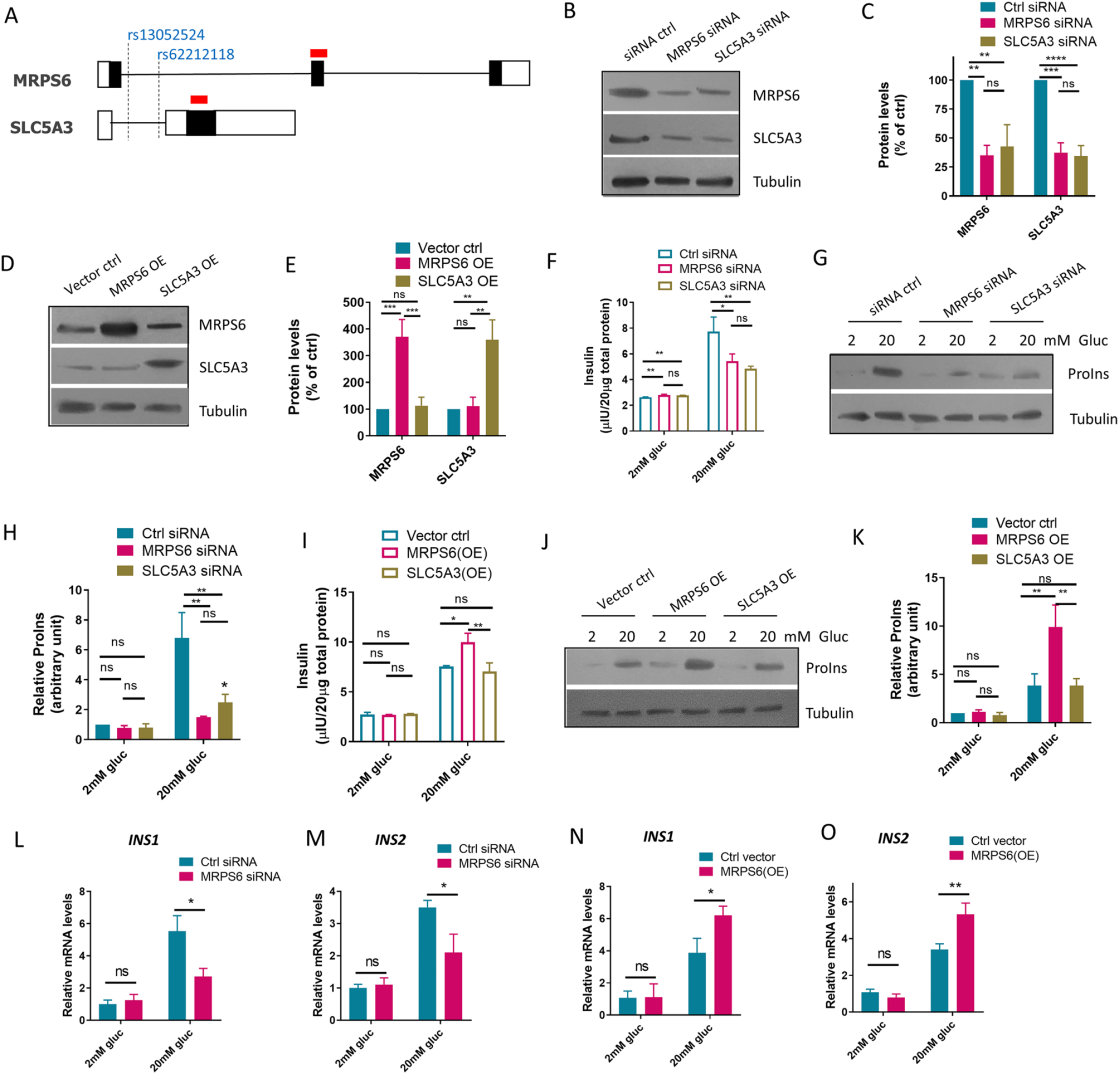
MRPS6 enhances glucose-stimulated insulin secretion (GSIS) in β-cells. (**A**) Schematic diagram of two IGT-associated SNPs on two overlapping genes: MRPS6 and SLC5A3. Lines indicate introns and boxes show exon, with empty boxes showing untranslated region (UTR) and black boxes showing protein coding region. Red bars indicate the region targeted by siRNAs. (**B**–**C**) siRNAs indiscriminately down-regulated the protein levels of both MRPS6 and SLC5A3. The insulinoma β-cells were transfected with siRNAs for 72 h and protein levels were examined by Western blotting and quantified by ImageJ. (**D**–**E**) MRPS6 and SLC5A were ectopically overexpressed in the insulinoma β-cells. Cells were transfected with plasmid bearing a cDNA of MRPS6 or SLC5A3 driven by CMV promoter for 72 h. Protein levels were examined by Western blotting. (**F**–H) MRPS6 or SLC5A3 knockdown reduced GSIS. The insulinoma β-cells were treated with siRNA as in (**B**) and GSIS was performed. Secreted insulin levels were quantified by ELISA (**F**) and intracellular proinsulin levels were examined by Western blot (**G**–**H**). (**I**–**K**) MRPS6 but not SLC5A3 overexpression enhanced GSIS. Overexpression was achieved as in (**D**). Secreted insulin levels were quantified by ELISA (**I**) and intracellular proinsulin levels were examined by Western blot (**J**–**K**). Shown immunoblots are representative data. (**L**–**O**) Glucose-induced insulin genes gene expression was downregulated and upregulated, respectively, by MRPS6 knockdown (**L**–**M**) and overexpression (**N**–**O**). Glucose stimulation was performed as in (**F**) and *INS1* and *INS2* mRNA levels were quantified by RT-qPCR. All experiments were performed > 3 biological repeats and error bars show standard deviation (SD) of the mean. *P* values are based on One-way ANOVA Turkey’s multiple comparison test or Student’s t-test (**L**–**O**): ns denotes not significant, *denotes *P* < 0.05, **denotes *P* < 0.01, ***denotes *P* < 0.001, ****denotes *P* < 0.0001.

Our previous GWAS has identified two SNPs that are associated with IGT in a cohort of east Asian in Hainan Province, China^18^. The two SNPs, rs62212118 and rs13052524, are located closely in the first intron of two overlapping MRPS6 and SLC5A3 genes (Fig. 1A). We therefore tested if the expression of affected genes was important for insulin secretion in a mouse insulinoma β-cells line. This cell line was generated using the same method described before^19^. MRPS6 and SLC5A3 genes overlap in the same region as in humans (Fig. 1A). We generated siRNAs targeting the coding region of the genes. However, both siRNAs downregulated the MRPS6 and SLC5A3 indiscriminately (Fig. 1B and C), preventing specific knockdown of the individual genes. The reason was unknown but could involve silencing/blockage of the overlapping 5’-UPR by mRNA cleavage products, similar to transitive/secondary siRNA in plants and *C. elegans*^20^. To differentiate the effect of MRPS6 and SLC5A3, we overexpressed the cDNA of both genes in β-cells (Fig. 1D and E). We then performed GSIS assay according to a well-established protocol^21^. Briefly, the β-cells were first starved in low glucose (2 mM) for 2 h then stimulated with high glucose (20 mM) for 2 h. Secreted insulin in the cell culture medium were measured with Enzyme-linked immunosorbent assay (ELISA) and intracellular proinsulin levels were measured by Western blot. Interestingly, MRPS6/SLC5A3 siRNA knockdowns reduced both secreted insulin levels (Fig. 1F) and intracellular proinsulin levels (Fig. 1G–H), indicating an impaired GSIS. Importantly, overexpression of MRPS6 but not SLC5A3 enhanced GSIS in the β-cells (Fig. 1I–K), suggesting that MRPS6 positively regulates GSIS. The mRNA levels of INS1 and INS2 were stimulated by glucose, however, such stimulation was downregulated by MRPS6 knockdown and further upregulated by MRPS6 overexpression, indicating that the effect on GSIS is at least partly attributed to MRPS6 regulation of insulin gene transcriptions.

### MRPS6 expression is positively correlated with UPR^mt^ marker genes in human pancreas

**Figure 2:**
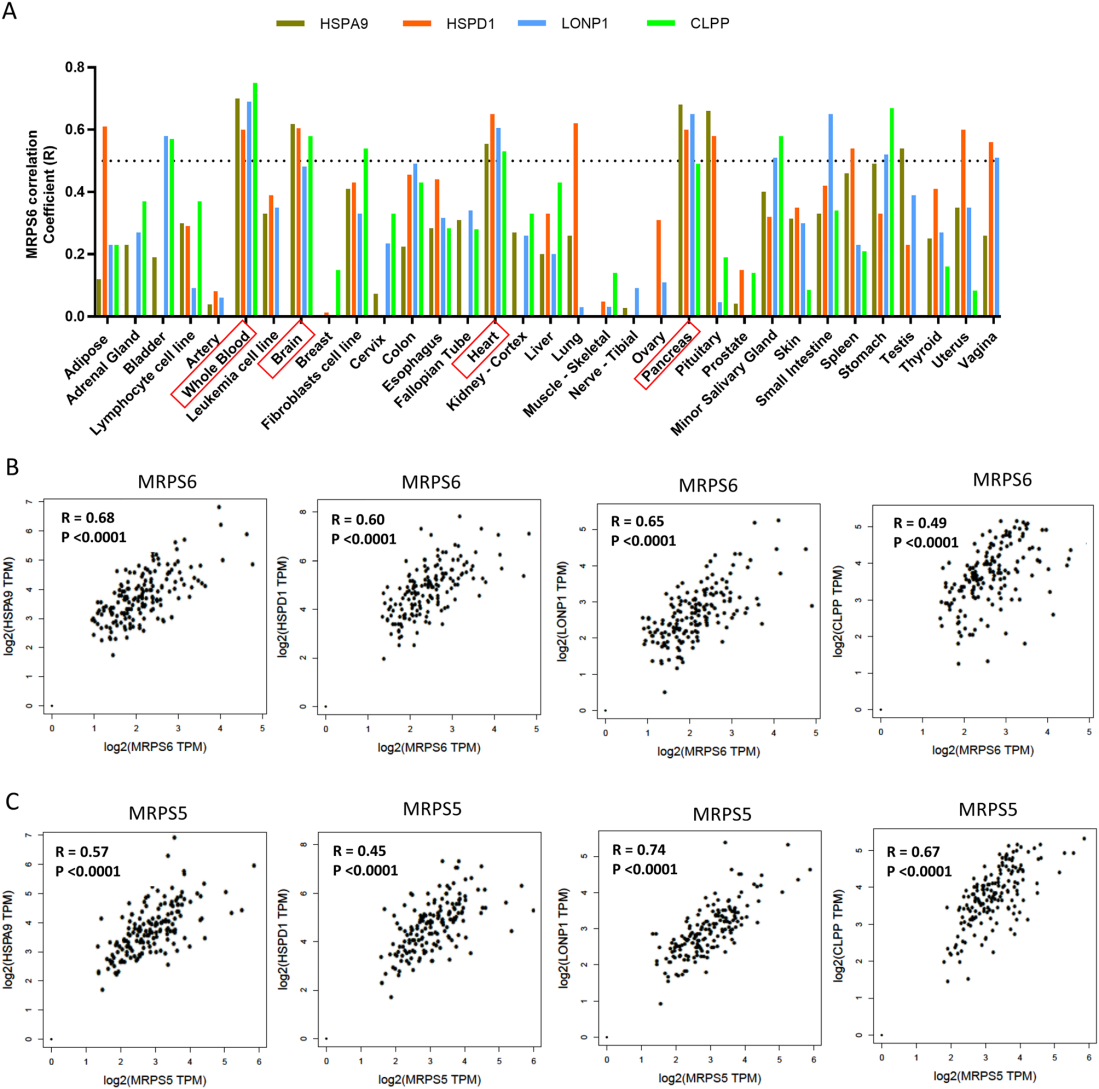
MRPS6 expression is positively correlated with UPR^mt^ marker genes in human pancreas. (**A**) Human MRPS6 expression was strongly associated with the expression of UPR^mt^ marker genes *HSPA9* (encoding mtHsp70), *HSPD1* (encoding Hsp60), *LONP1* (encoding LonP1), and *CLPP* (encoding ClpP) in specific tissues. Graphs were generated by plotting Pearson correlation efficient generated from GEPIA website using datasets from human pancreas tissues in the GETx database. R stands for Pearson correlation coefficient indicating the degree of association: “0” denotes no association and “1” denotes perfect positive association. Highlighted in red boxes are tissues (blood, brain, heart, and pancreas) with R around or higher than 0.5. (**B**) Correlation plots of the expression of MRPS6 and UPR^mt^ marker genes. (**C**) Correlation plots of another mitochondrial ribosome gene MRPS5 and UPR^mt^ marker genes.

MRPS6 encodes a subunit of mitochondrial ribosome. Another subunit MRPS5 has been known to regulate UPR^mt^ in *C. elegans* and mice through protein imbalance between mitochondria and nucleus^22^. We reasoned that MRPS6 could also regulate UPR^mt^ to affect GSIS. First, we checked if the expression of MRPS6 was correlated to UPR^mt^ genes in various human tissues by analyzing the Genotype-Tissue Expression (GTEx) database. This database consists of transcriptomic profiles of > 15,000 samples from 54 tissues of over 800 donors, which is a valuable resource for studying the genetics of human disease^23^. We analyzed the data with a broadly used informatics platform: Gene Expression Profiling Interactive Analysis (GEPIA)^24^. The results showed that expression of MRPS6 was in general positively correlated with UPR^mt^ gene expression in most tissues (Fig. 2A, a full panel in Supplemental Figure S1). Interestingly, correlation was especially strong in whole blood, brain, heart, and pancreas, with Pearson coefficient around 0.5 or higher for all the 4 UPR^mt^ marker genes (*HSPA9*, *HSPD1*, *LONP1*, and *CLPP*). In pancreas, the correlation between MRPS6 and *HSP9A*, *HSPD1*, *LONP1* and *CLPP* was highly significant (*P* < 0.0001) and strong (R = 0.68, 0.60, 0.65, and 0.49, respectively) (Fig. 2B). We compared the correlation of MRPS6 and MRPS5 with UPR^mt^ (Fig. 2C). Consistently, expression of MRPS5 was also strongly correlated with UPR^mt^ in pancreas, suggesting that UPR^mt^ is active in pancreas. There was no or weak correlation with liver, muscle, and adipose tissues (Fig. 2A), suggesting a specific role in the pancreas.

### MRPS6 knockdown activates UPR^mt^ through negative feedback mechanisms

**Figure 3:**
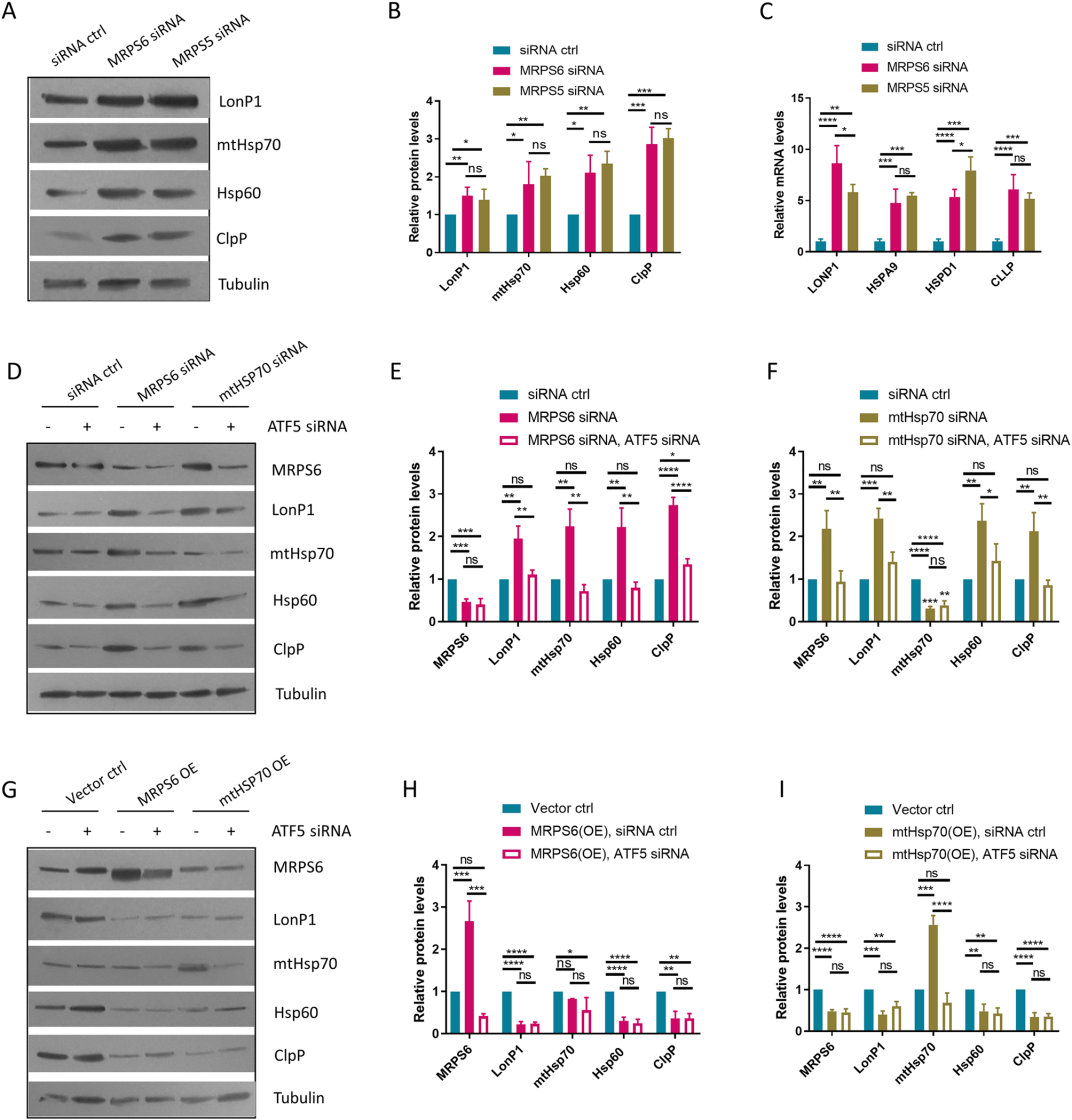
MRPS6 knockdown activates UPR^mt^ through negative feedback mechanisms. (**A**–**C**) MRPS6 and MRPS5 siRNAs increased the expression of UPR^mt^ markers (LonP1, mtHsp70, Hsp60, and ClpP). The insulinoma β-cells were transfected with siRNAs for 72 h and protein levels were examined by Western blotting (**A**) and quantified (**B**). mRNA levels of UPR^mt^ target genes (*LONP1*, *HSPA9*, *HSPD1*, and *CLPP*) were quantified by RT-qPCR (**C**). (**D**–**F**) MRPS6 and mtHsp70 knockdowns increased the expression of each other and UPR^mt^ makers (LonP1, mtHsp70, Hsp60, and ClpP), which were dependent on ATF5. The insulinoma β-cells were transfected with indicated siRNAs for 72 h and protein levels were examined by Western blotting (**D**) and quantified (**E**–**F**). (**G**–**I**) MRPS6 and mtHsp70 overexpression reduced the expression of UPR^mt^ marker genes. The insulinoma β-cells were transfected with indicated siRNAs and expression plasmids for 72 h and protein levels were examined by Western blotting (**G**) and quantified (**H**–**I**). Shown immunoblots are representative data. All experiments were performed > 3 biological repeats and error bars show standard deviation (SD) of the mean. *P* values are based on One-way ANOVA Turkey’s multiple comparison test: ns denotes not significant, *denotes *P* < 0.05, **denotes *P* < 0.01, ***denotes *P* < 0.001, ****denotes *P* < 0.0001.

The correlation between MRPS6 and UPR^mt^ suggests a positive regulatory mechanism. We asked if MRPS6 knockdown would impair UPR^mt^. Surprisingly, however, MRPS6 siRNA treated β-cells showed elevated protein levels of UPR^mt^ markers (mtHSP70, HSP60, LonP1, ClpP), indicative of a negative role (Fig. 3A–B). The negative regulation of UPR^mt^ by MRPS6 was confirmed by real-time quantitative polymerase chain reaction (RT-qPCR), showing that mRNA levels of UPR^mt^ markers were robustly elevated by MRPS6 siRNA (Fig. 3C). In cell signal transduction, it is not uncommon that expression of downstream genes feedback to upstream regulators to fine tune the signaling^25^. Indeed, knocking down of *C. elegans* genes *hsp-6* (mtHSP70 homolog) or *hsp-60* (HSP60 homolog) can activate UPR^mt^^26^. Consistent with the *C. elegans* studies, like MRPS6 knockdown, mtHsp70 knockdown also activated UPR^mt^ (Fig. 3D–F). Importantly, like other UPR^mt^ maker genes, MRPS6 expression was increased by mtHsp70 knockdown (Fig. 3F), suggesting that it is positively regulated by UPR^mt^.

To confirm the role of MRPS6 in UPR^mt^ regulation, we knocked down the key transcription factor ATF5 in addition to MRPS6 and mtHSP70 knockdowns. The results showed that the activation of UPR^mt^ by the latter two modulations was largely dependent on ATF5, as the increase in UPR^mt^ gene expression was dampened by ATF5 siRNA (Fig. 3D–F). Consistent with a negative feedback mechanism, overexpression of MRPS6 or mtHsp70 inhibited UPR^mt^, which was also dependent on ATF5 (Fig. 3G–I). Therefore, like mtHsp70, MRPS6 is positively regulated by UPR^mt^ while its expression can negatively feedback to inhibit UPR^mt^.

### UPR^mt^ negatively regulates GSIS

**Figure 4:**
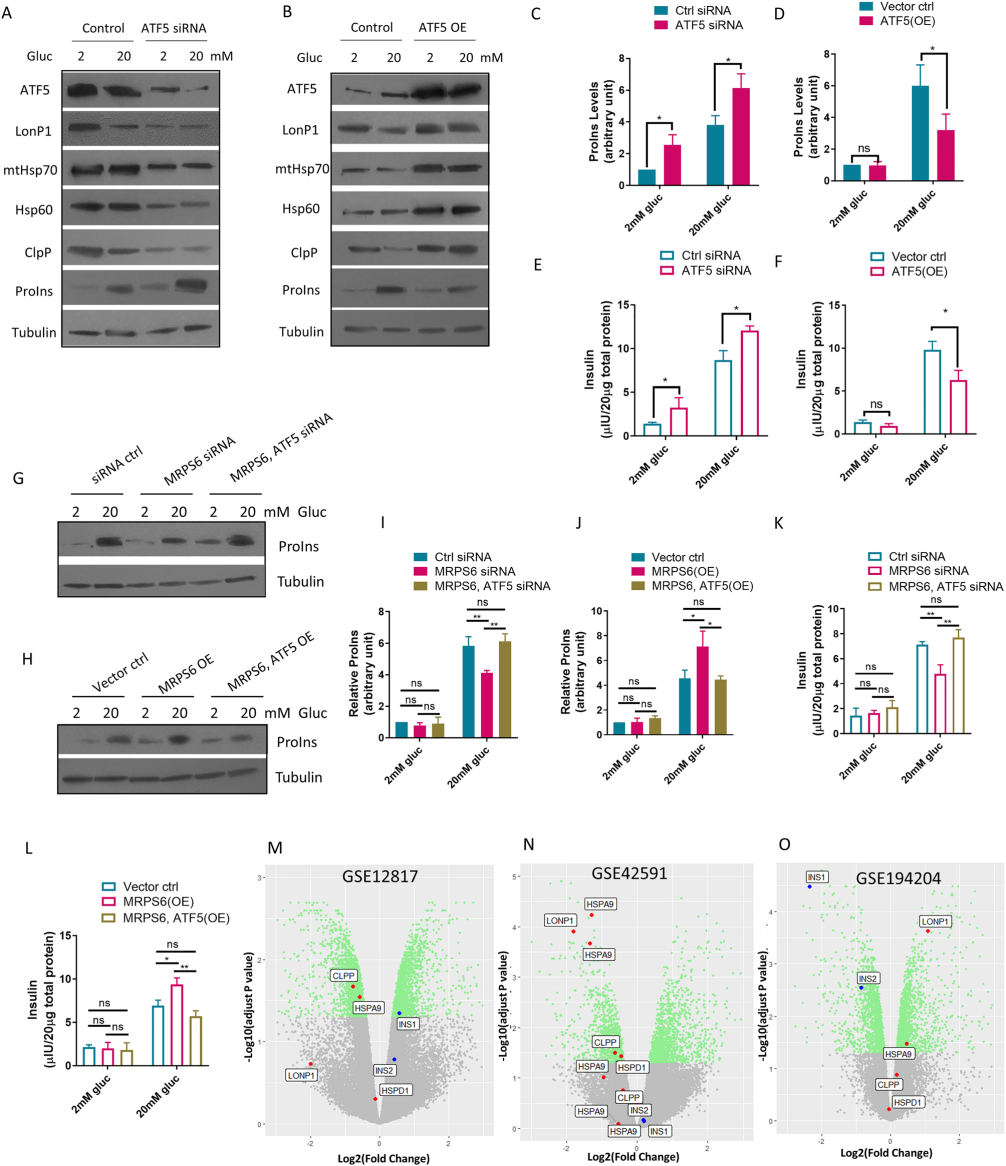
UPR^mt^ negatively regulates GSIS in β-cells. (**A**–**D**) ATF5 siRNAs and overexpression (OE) increased and decreased, respectively, the intracellular proinsulin (ProIns) levels in high glucose conditions. The insulinoma β-cells were transfected with siRNA or overexpression plasmid for 72 h and GSIS was performed. Cells were collected for Western blotting of ProIns levels and UPR^mt^ makers LonP1, mtHsp70, Hsp60, and ClpP (**A**–**B**) and quantified (**C**–**D**). (**E**–**F**) ATF5 siRNAs and overexpression (OE) increased and decreased secreted insulin levels. The insulinoma β-cells were treated as in (**A**–**B**) then subject to GSIS assay. Secreted insulin levels in cell culture were quantified by ELISA. (**G**–**K**) Effect of MRPS6 on GSIS was dependent on ATF5. The insulinoma β-cells were transfected with siRNA (**G**) or overexpression plasmid (**H**) for 72 h. ProIns levels were examined by Western blotting (**I**–**J**). Secreted insulin levels were quantified by ELISA (K-L). (**M**–**O**) UPR^mt^ genes (red data points) and insulin genes (blue data points) were negatively correlated in mouse transcriptomic studies. Rat (**M**) or mouse (**N**) islets were isolated, treated with low and high glucose, then subject to microarray analysis. Islets were isolated from MFN1 and MFN2 deletion and WT mice then subject to RNA sequencing analysis (**O**). All data in bar graphs were from > 3 experimental repeats and error bars show standard deviation (SD) of the mean. *P* values are based on Student’s t-test (**C**–**D**, **E**–**F**) or One-way ANOVA Turkey’s multiple comparison test (**I**–**L**): ns denotes not significant, *denotes *P* < 0.05, **denotes *P* < 0.01, ***denotes *P* < 0.001, ****denotes *P* < 0.0001.

The regulatory interaction between MRPS6 and UPR^mt^ promoted us to test if UPR^mt^ would participate in GSIS in the β-cell line. By knocking down the UPR^mt^ transcription factor ATF5 expression, which effectively down-regulated UPR^mt^ marker genes including LonP1, mtHsp70, Hsp70, and ClpP (Fig. 4A, quantification in Supplemental Figure S2A), we found that impaired UPR^mt^ increased GSIS, as demonstrated by increased proinsulin levels in response to high glucose (20 mM) media (Fig. 4A and C). Conversely, activation of UPR^mt^ by ATF5 overexpression, evidenced by up-regulation of UPR^mt^ marker genes (Fig. 4B, quantification in Supplemental Figure S2B), reduced glucose-stimulated proinsulin levels (Fig. 4B and D). Consistently, extracellular mature insulin levels were increased by ATF5 knockdowns and impaired by ATF5 overexpression (Fig. 4E–F). These results demonstrated that UPR^mt^ was important for GSIS. We further asked if UPR^mt^ was required for MRPS6 regulation of GSIS shown in Fig. 1 by siRNA knocking down or overexpressing MRPS6 and ATF5 simultaneously. Consistent with results in Fig. 1F–K, MRPS6 knockdown reduced glucose-stimulated proinsulin (Fig. 4G and I) and mature insulin levels (Fig. 4K), however, further knocking down ATF5 largely restored the GSIS. Conversely, overexpression of MRPS6 enhanced glucose-stimulated proinsulin levels (Fig. 4H and J) and mature insulin levels (Fig. 4L) but such enhancement was largely prevented by further ATF5 overexpression. Together, these experiments consistently suggest that MRPS6 regulates GSIS through UPR^mt^.

### UPR^mt^ is negatively correlated with insulin gene expression in mouse transcriptomes

We wondered if the suppressing role of UPR^mt^ in GSIS could be true in vivo. We analyzed transcriptomes of human or mouse islets, hoping to find evidence supporting our β-cell line studies. We reasoned that if UPR^mt^ negatively regulated GSIS, insulin gene expression would be negatively correlated with UPR^mt^. However, analysis of GETx database showed that there was no correlation of insulin gene expression with that of UPR^mt^ (Supplemental Figure S3). Since these donated islets could be affected by different illnesses leading to the end of life and various treatment received surrounding the end of life, their insulin and UPR^mt^ gene expression could be highly variable. We therefore analyzed mouse transcriptomes under conditions known to stimulate or downregulate insulin gene expression. Gene Expression Omnibus (GEO) is a database repository of high throughput gene expression data and hybridization arrays, chips, microarrays^27,28^. In one microarray study (GSE12817), isolated rat islets were treated with 2-, 5-, and 10-mM glucose for 18 h^29^. We compared the transcriptomes of 2 mM and 10 mM glucose treatments. As shown in the volcano plot in Fig. 4M, insulin gene INS1 and INS2 expression (blue data points) were increased by high (10 mM) versus low (2 mM) glucose as expected. Interestingly, the expression of UPR^mt^ marker genes (red data points) including *HSPA9*, *HSPD1*, *LONP1*, and *CLPP* were all downregulated, with *CLPP* and *HSPA9* passed the *P* < 0.05 threshold (green). In another microarray study (GSE42591) treating mouse isolated islets with increasing concentrations of glucose^30^. We compared high glucose group (11 and 16 mM) with low glucose group (3 and 5 mM) and found that, consistently, high glucose suppressed UPR^mt^ gene expression (Fig. 4N). Multiple DNA probes showed differential expression levels for the HSPA9 and CLPP, but they are all consistently downregulated (Fig. 4N, red data points), with at least one of the probes for each UPR^mt^ markers reaching statistical significance (*P* < 0.05). Additionally, in a RNA sequencing dataset (GSE194204) of mouse islets lacking mitochondrial fusion (MFN1 and MFN2 double deletion)^31^, insulin expression was suppressed (blue data points) but UPR^mt^ marker gene expression was increased (Fig. 4O, red data points). Therefore, the negative correlation of UPR^mt^ with insulin expression is likely true in mouse islet cells, lending further support to our observations in the insulinoma β-cells.

### UPR^mt^ mediates MRPS6 regulation of glucose-induced ROS and apoptosis

**Figure 5:**
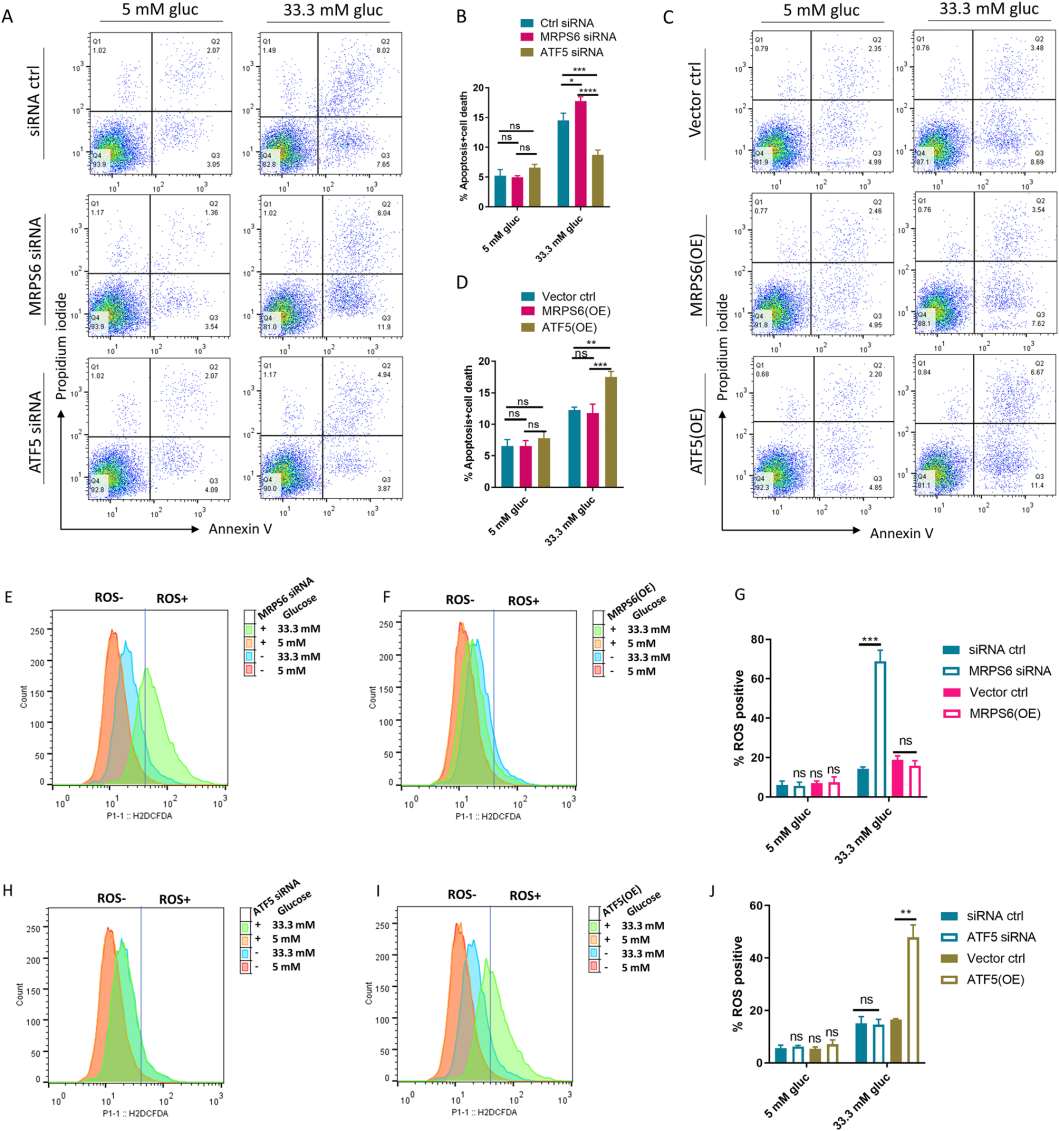
UPR^mt^ mediates MRPS6 regulation of glucose-induced ROS and apoptosis. (**A**–**B**) Knocking down MRPS6 and ATF5 increased and decreased, respectively, the apoptosis and cell death of insulinoma β-cells. Cells were transfected with siRNA for 72 h and treated with 5 mM or 33.3 mM glucose (gluc) for 24 h. Apoptosis and cell death were measured by Annexin V and propidium iodide in flow cytometry. (**C**–**D**) Overexpressing (OE) ATF5 increased the apoptosis and cell death in insulinoma β-cells. Cells were transfected with ATF5-overexpression plasmid for 72 h and treated with 3 mM or 33.3 mM glucose for 24 h. Apoptosis and cell death were measured and quantified as in (**A**–**B**). (**E**–**J**) MRPS6 knockdown and ATF5 overexpression increased intracellular ROS in β-cells . siRNA knockdown, overexpression (OE), and glucose treatment were performed as in (**A**–**D**). ROS levels were measured by staining cells with DCFH-DA and flow cytometry. All data in bar graphs were from > 3 experimental repeats and error bars show standard deviation (SD) of the mean. *P* values are based on One-way ANOVA Turkey’s multiple comparison test (**B**, **D**) or Student’s t-test (**G**, **J**): ns denotes not significant, *denotes *P* < 0.05, **denotes *P* < 0.01, ***denotes *P* < 0.001, ****denotes *P* < 0.0001.

Hyperglycemia can induce apoptosis and cell death in insulin secreting cells, contributing to IGT and T2D development^32,33^. We asked if MRPS6 and UPR^mt^ could play a role in pancreatic cell apoptosis. We treated the insulinoma β-cells for 24 h with normal (5 mM) and high (33.3 mM) glucose known to induce apoptosis^34^. This concentration was chosen over 20 mM glucose used in other experiments because the latter gave more variable results. Then we modulated the gene expression by siRNA knockdown (Fig. 5A–B) and overexpression (Fig. 5C–D) and examined apoptosis (Annexin V), cell death (Propidium iodide), and ROS marker (DCFDA) by flow cytometry. As the results shown in Fig. 5A–B, high glucose induced significant amount of apoptosis and cell death. Interestingly, MPRS6 knockdown further increased apoptosis and death in the presence of 33.3 mM glucose but had no effect on cells growing at 5 mM glucose conditions (Fig. 5B). To the opposite, ATF5 knockdown decreased glucose-induced apoptosis and cell death (Fig. 5A–B), suggesting a negative function of UPR^mt^ on cell survival. Consistently, in overexpression (OE) experiments, UPR^mt^ activation by ATF5 OE significantly enhanced apoptosis and cell death (Fig. 5C–D). MRPS6 OE had no effect on cell death and apoptosis, suggesting that MRPS6 OE is not sufficient to regulate β-cell apoptosis. In ROS measurement, apoptosis and cell death were much higher in 33.3 mM glucose medium as compared to 5 mM glucose medium. MRPS6 siRNA knockdown further increased ROS levels in high but not low glucose conditions (Fig. 5E and G), in line with the effect on cell death (Fig. 5A–B). MRPS6 OE did not change the ROS levels in response to high glucose (Fig. 5F). ATF5 OE enhanced ROS production under high glucose conditions but ATF5 siRNA knockdown did not show an effect (Fig. 5H–J). Together, our experiments uncovered an important role of MRPS6 and UPR^mt^ in regulating apoptosis and cell death in β-cells.

## Discussion

Our previous work has identified two IGT-associated SNPS (rs62212118 and rs13052524) located at the first intron of two overlapping genes: MRPS6 and SLC5A3^18^. In this study, we modulated the expression of MRPS6 and SLC5A3 and found that MRPS6 but not SLC5A3 was involved in GSIS in mouse pancreatic β-cells. Further analysis of MRPS6 has led to the discovery of UPR^mt^ in repressing GSIS. UPR^mt^ also enhanced glucose-induced ROS and apoptosis, suggesting an important role of UPR^mt^ in the development of IGT and T2D. Different from a previous studying showing that ATF5 promotes β-cell survival^16^, our study finds that ATF5-mediated UPR^mt^ increases β-cell apoptosis in response to high glucose stimulation (Fig. 5). As we did not observe significant effects of ATF5 on cell survival under normal glucose conditions, the opposing roles of ATF5 could simply result from different glucose concentrations.

Mitochondrion is the center for glucose and fatty acid metabolisms and has been heavily implicated in T2D^10,11^. The underlying molecular mechanisms remain poorly understood. Impaired mitochondria are known to generate excessive ROS, induce cell apoptosis, and compromise glucose and lipid metabolisms, all of which have been linked to the pathology of T2D^35,36^. Interestingly, a large category of diabetes, termed mitochondrial diabetes, can be caused by mitochondrial DNA (mtDNA) mutations or deletions^37,38^. The causality of abnormal mtDNA can be determined because the disease follows strictly a maternal inheritance pattern. Strikingly, many of these DNA abnormalities affect tRNA genes, including m.3243A > G mutation in tRNA-Leu, m.8344 A > G mutation in tRNA-Lys, and m.14709 T > C mutation in tRNA Glu^38^. These suggest that protein translation in the mitochondria plays important roles in IGT and T2D. The identification of MRPS6 and UPR^mt^ in our study strengthens this idea and provides further details on the roles of mitochondria in T2D development.

UPR^mt^ is a mitochondrial stress response that is proposed to be induced by imbalanced protein synthesis between mitochondrion and nucleus, which in turns controls gene expression in the nucleus to balance the protein homeostasis^14,22^. Impaired UPR^mt^ has recently been shown to be important for a variety of disease including cancer and neurodegenerative diseases, but its role in IGT and T2D remains unclear^17,39^. Interestingly, however, the current paradigm proposes UPR^mt^ as a rescue response which plays a beneficial role in preventing disease and extending lifespan. In contrast, our study uncovers a negative role of UPR^mt^ in T2D. It is not uncommon that a stress response pathway could function opposingly depending on different contexts. In addition, the paradox could also be due to different phenotypes examined. For example, on the one hand UPR^mt^ could boost the functions of mutated mitochondria to favor survival, therefore mitigate apoptosis-related disease, on the other hand, however, this process could accumulate mutated mitochondria otherwise destroyed by autophagy or cell death, leading to metabolic diseases in the long term. Indeed, a study in *C. elegans* shows that UPR^mt^ could promote the amplification of impaired mtDNA, leading to reduced mitochondrial function^40^. Similarly, in our case, the reduction in GSIS could be resulted from the pro-survival functions of UPR^mt^ at the expense of keeping unfit mitochondria. It would be interesting to ask if T2D and other UPR^mt^-affected diseases are indeed attributed to the “beneficial” functions of UPR^mt^ in promoting cell survival.

It is interesting that the association between MRPS6 and UPR^mt^ prefers only a few human organs (brain, blood, heart, and pancreas). MRPS6 is a mitochondrial ribosome subunit important for proper translation of mtDNA. The preferential correlations suggest that these organs are more dependent on mitochondrial translation. Indeed, brain and heart consume more energy than other organs^41^, therefore require tighter control over energy metabolism. This would predict that malfunctions of MRP6 and UPR^mt^ would preferentially affect these organs. Indeed, MRPS6 SNPs have been reported to be associated with heart disease^42,43^. Consistently, mutations in mitochondria-specific tRNA also preferentially affect neuronal systems and cardiovascular systems^44^. Although the pancreas is not traditionally viewed as an energy demanding organ, insulin release relies on high energy (glucose) stimulation. Therefore, it is conceivable that pancreas would require tight regulation of mitochondrial metabolisms under high glucose conditions. This is consistent with our experimental results showing that MRPS6 and UPR^mt^ knockdown or overexpression usually affect GSIS and apoptosis only under high glucose conditions.

The correlation analysis in Fig. 2 is based on human pancreas, of which only 3–5% are β-cells. We therefore also asked if such correlation was true in β-cells. GEO dataset GSE124742 is a recent single cell RNAseq study of islet from over 31 donors^45^. We selected β-cells based on the well-established marker INS and examined the correlation of MRPS6 with UPR^mt^ marker genes. Consistent with whole pancreas study in Fig. 2, MRPS6 was positively correlated with UPR^mt^ with statistical significance (Supplemental Figure S4). The Pearson coefficients were not as strong as those in Fig. 2. We believe it is hard to make an accurate comparison because of the following reasons. First, GTEx data in Fig. 2 has over 100 donors while GSE124742 has only 31 donors; donor variations are general big and the limit donor number in GSE124742 could reduce the statistical robustness. Second, the GTEx samples are collected overtime with different methods while the GSE124742 data here exemplify only 1 experiment; the Pearson coefficient in these two scenarios cannot be quantitatively compared. Nevertheless, the positive correlation in human β-cells supports our wet lab studies in mice counterpart.

To further corroborate the idea that the regulation of UPR^mt^ by mRPS6 affect β-cell, we took effort to repeat a few essential experiments using primary β-cells. We confirmed the β-cell purify ~ 90% by flow cytometry analysis of β-cell marker insulin and α-cell marker glucagon (Figure S5A). The cells were transiently transfected with mRPS 6 siRNA then the mRNA levels of UPRmt marker gene was quantified by RT-qPCR. GSIS assay was also performed. As shown in Figure S5B–C), mRPS6 knockdown consistently increased UPR^mt^ gene expression (Figure S5B). Also consistent with the insulinoma β-cell study, mRPS6 siRNA significantly reduced GSIS of primary mouse β-cells by ELISA (Figure S5C). An additional interesting question we addressed is whether mRPS6 or ATF5 knockdown or overexpression would affect the processing of proinsulin to insulin. We used an antibody against mature insulin, which also recognized proinsulin, and showed that no significant changes were observed for the proinsulin/insulin ratio, suggesting that the processing and maturation of proinsulin was not controlled by mRPS6 or ATF5 (Figure S6).

How the IGT-associated SNPs regulate MRPS6 expression remains to be investigated. It is common that SNPs in the intron region regulate gene expression through transcription efficiency, nuclear export, transcript stability, splicing efficiency, or alternative splicing^46^. As MRPS6 knockdown and overexpression in β-cells decreased and increased GSIS, respectively, we propose that the SNPs likely function to reduce MRPS6 expression. However, as many SNPs located in the intron regions have no roles in regulating nearby gene expression, further studies are needed to confirm our results.

Our study did not rule out a potential function of SLC5A3 in IGT. First, as it is not possible to knock down SLC5A3 expression without reducing MRPS6 expression, we have relied on overexpression to suggest that SLC5A3 is not likely involved in GSIS. However, since SLC5A3 may require additional layers of regulation such as posttranslational modifications, membrane sequestration, or ligand-dependent activation, overexpression would not be sufficient to affect GSIS. Second, it remains possible that SLC5A3 functions in the insulin targeting tissues such as adipose or muscle to affect insulin sensitivity. Additionally, the implication of MRPS6 in GSIS does not exclude the involvement of SLC5A3, as although rare, genes affected by the same SNP could simultaneously contribute to a disease phenotype. Additional studies are required to clarify the role of SLC5A3 in IGT and diabetes.

Another limitation of our study is that we mostly used the insulinoma cell line for causal study. As in vitro cultured β-cells are heterogeneous and could loss GSIS potential with extensive passages, studies with in vitro beta cell culture will need to be confirmed with in vivo study, ideally by manipulating UPR^mt^ in animal models and examining T2D phenotypes. Nevertheless, we have been able to obtain supporting evidence from analyzing public datasets from RNAseq and microarray that are practically feasible and statistically robust. By analyzing two microarray datasets with mouse islets treated with various glucose concentrations and one RNA sequencing dataset where insulin expression was suppressed by impaired mitochondrial fusion, we show that there is a negative correlation between insulin and UPR^mt^ expression. Despite the fact that no causal relationship can be derived from the data analysis, the work has lent strong support to our in vitro cell culture data and encourage future in vivo studies.

## Methods

### Cell culture and transfection

The insulinoma β-cell line was established from a transgenic female C57BL/6 mouse expressing SV40 large T antigen under the control of human insulin gene promoter. The cell line used in this study is derived from a female mouse and retained robust GSIS until passage 40. The insulinoma β-cells were maintained in DMEM containing 25 mM glucose, 10% fetal bovine serum, 50 μM β-mercaptoethanol, in a humidified incubator at 37 °C and 5% CO_2_. siRNAs or overexpression plasmids were delivered into cells for transient knockdown or overexpression using HiPerfect transfection reagent (Qiagen). Briefly, fresh cells at 60–90% confluency was trypsinized and diluted in complete media to 5E4 cells/ml. For each well of 96-well plate, 5 pmol of siRNA was diluted in 25 ml OptiMEM (Invitrogen) and 0.75 ul of HiPerfect reagent was added and mixed, incubated at room temperature for 5 min. 175 ul cells were then added to each well. Cells were grown at 37 °C in humid incubator for 72 h and gene expression was examined by qPCR or Western blotting. The sequence of PCR primers and siRNA oligos can be found in the Supplemental material Table S1.

### Real-time quantitative PCR

Cells were washed with PBS 3 times total RNA was extracted with TRIzol Reagent (Invitrogen) according to manufacturer’s manual. RNA was washed with 70% ethanol and suspended in RNase-free H_2_O. RNA was reverse transcribed by using HiScript II Q RT SuperMix for qPCR (Vazyme) according to provider’s manual. RT-qPCR was performed in T100 Thermal Cycler (Bio-Rad) using AceQ Universal SYBR qPCR Master Mix (Vazyme). mRNA levels were normalized to that of GAPDH. Changes in the transcript level were calculated using the 2^−ΔΔCT^ method. Primer sets can be found at Supplemental materials Table S2.

### Western blotting

Cells were rinsed with ice-cold PBS 2 times, then lyzed with RIPA buffer (10 mM Tris–HCl, pH 8.0, 1 mM EDTA, 0.5 mM EGTA, 1% Triton X-100, 0.1% Sodium Deoxycholate, 0.1% SDS) by shaking at 4 °C for 20 min. Cell lysate were centrifuged and total protein in the supernatant were determined with BCA protein kit. For all samples, equal amounts of proteins were subject to SDS-PAGE then transferred to PVDF membrane. The membranes were blocked in 5% non-fat milk for 1 h then probed with primary antibodies in 5% non-fat milk for 1 h. The membranes were washed with PBST (PBS + 0.5% Tween-20) for 5 times, 10 min each time, then incubated with HRP-conjugated secondary antibodies. After extensive washing as above, the membranes were developed by enhanced chemiluminescence (ECL). The uncropped immunoblots were shown in Figure S7 in Supplemental Information.

### Glucose stimulated insulin secretion (GSIS)

The insulinoma β-cells were sub-cultured with no more than 5 passages to keep their GSIS potential. GSIS assay was performed similarly to a previous report^21^ with slight modifications. Cells were cultured in DMEM with 25 mM glucose to 80% confluency, then washed 3 times with low glucose buffer (25 mM HEPES pH 7.4, 125 mM NaCl, 6 mM KCl, 1.2 mM MgCl_2_, 1.3 mM CaCl_2_, 2 mM), maintained in the low glucose buffer for 30 min, washed 3 times with low glucose buffer again, and maintained at low glucose buffer for 2 h. Cells were then changed to (1) low glucose buffer as control and (2) high glucose buffer (same buffer except with 20 mM glucose) and maintained for 2 h. Equal volumes of the incubation buffer were removed and centrifuged at 20,000 RFC to collect supernatant for ELISA. Attached cells were washed with PBS buffer then lysed with RIPA buffer. Protein concentration was measured with BCA protein assay kit. Equal amounts of total protein were subject to Western blotting for proinsulin levels.

### Enzyme-linked immunosorbent assay (ELISA)

Collected cell supernatants were first adjusted according to total protein extracted from the same well so as to make sure they are released from the same number of cells. ELISA was performed using Insulin Mouse ELISA Kit (Catalog # EMINS) according to product instructions. Briefly, cell supernatant and standard were coated on the ELISA plate overnight at 4 °C, then washed Wash Solution (300 μl) for 3 times. Wash solution was then removed by inverting the plate and blot it against clean paper towels before a biotinylated insulin was added at the instructed concentration. After 1 h incubation at room temperature, the wells were washed extensively with Wash solution again and 100 μl of 1X HRP-Streptavidin was added to each well and incubated 45 min at room temperature. After another round of extensive washing, 100 μl of TMB reagent was added to each well and incubated for 30 min at room temperature for color development. The reaction was then stopped by adding 50 μl of Stop Solution and read at 450 nm immediately. The insulin concentration in the samples were determined by using a standard curve generated from the standard.

### Apoptosis and cell death analysis

Cells were treated with 33.3 mM glucose for 24 h to induce apoptosis as reported before^34^. Flow cytometry was used for apoptosis analysis as reported before^47^. In brief, attached cells were harvested by trypsin treatment and washed with cold PBS containing 0.5% BSA (bovine serum albumin) for 3 times by centrifuge at 200 RFC. Cell epitopes were then blocked in PBS containing 0.5% BSA on ice for 1 h. Cells were centrifuged and resuspended in Annexin-binding buffer (10 mM HEPES pH 7.4, 140 mM NaCl, and 2.5 mM CaCl2) to 1E6 cells/ml. 5 µL of the Annexin V-FITC conjugate was added to 100-μl above sample and incubated for 15 min on ice. Cells were then washed with Annexin-binding buffer 3 times and resuspended the same buffer with 2 µM Propidium iodide (PI). Apoptosis and cell death were quantified using cytoFLEX S (BECKMAN). Data were analyzed and plotted by using FlowJo (V10.7) software.

### ROS measurement

Cells were washed with PBS containing 0.5% BSA, followed by incubation with 10 µM DCFH-DA in the same buffer at 37 °C for 30 min in the dark. Cells were then washed 3 times with PBS containing 0.5% BSA and resuspended in the same buffer. Cellular ROS contents were quantified by using cytoFLEX S (BECKMAN) flow cytometer.

### Data analysis, visualization, and statistics

Bar graphs were generated in GraphPad Prism software with error bars stand for standard deviation of the mean from 3 biological repeats. *P* values were obtained by two-tailed, unpaired Student’s t-test or One-way ANOVA turkey’s multiple comparison test using GraphPad Prism software. Statistical significance is as follows: ns denotes not significant, *denotes *P* < 0.05, **denotes *P* < 0.01, ***denotes *P* < 0.001. Human gene expression correlation graphs were generated by using GEPIA website R indicating Pearson’s correlation coefficient. GEO datasets were downloaded from NCBI website and analyzed by using Rstudio software loaded with limma packages. Volcano Plots were generated in Rstudio using ggplot2 package. Expression tensity in Western blots were quantified by using ImageJ software.

### Supplementary Information

Supplementary Information.

## Data Availability

The GETx data analyzed in this study could be found in GEPIA website: http://gepia.cancer-pku.cn. The RNA sequencing datasets and microarray datsets analyzed in this study can be found in NCBI website: https://www.ncbi.nlm.nih.gov/gds. All data generated and analysed during this study are included in this published article (and its Supplementary Information files).
