# Supplementary material for: MRPS6 modulates glucose-stimulated insulin secretion in mouse islet cells through mitochondrial unfolded protein response
Source: Sci Rep. 2023 Sep 27;13:16173. doi: 10.1038/s41598-023-43438-7 (PMC10533529; doi:10.1038/s41598-023-43438-7)
Supplement: Supplementary file 1 — Supplementary Information. [file 41598_2023_43438_MOESM1_ESM.pdf]

Supplemental Information

MRPS6 modulates glucose-stimulated insulin secretion in mouse islet cells through mitochondrial unfolded protein response

Danhong Lin[#], Jingwen Yu[#], Leweihua Lin, Qianying Ou and Huibiao Quan[*]

Department of Endocrinology, Hainan General Hospital, Hainan Affiliated Hospital of Hainan Medical University, Haikou, China

Supplemental Materials

β-cell line was purchased from Yeasen Biotechnology (Shanghai, China), which were originally isolated from transgenic insulinoma mouse model in Shanghai Jiao Tong University School of Medicine. This cell line was screened from multiple insulinoma beta cell lines derived from transgenic mice expressing SV40 large T antigen under the control of human insulin gene promoter. Briefly, SV40 large T antigen gene fused to human insulin promoter was microinjected to fertilized eggs of C57BL/6 mice and the offspring developing insulinoma were sacrificed obtain islets. Islets were then dissociated with enzymes and stable cell lines were selected. The cell lines were confirmed to be β cells by testing the expression of insulin genes and other markers of the β cell. The cell lines then were tested for glucose stimulated insulin secretion (GSIS) at passage 10, 20, 30, 40. 50, 60 to establish a stable model for in vitro β -cell. The cell line used in this study is derived from a female mouse and retained robust GSIS until passage 40. This β -cell line was authenticated by STR method by BeNa Culture Collection (report in Supplemental Information). Mouse primary β-cells were purchased from (Procell Life Science & Technology, Cat. CP-M200). DMEM medium and Fetal bovine serum (FBS) was purchased from Thermo Fisher Scientific (Waltham, MA, USA). Penicillin-streptomycin was purchased from Yeasen Biotechnology（Shanghai，China）siRNAs and qPCR primers (Table S1 and S2) were synthesized by GenePharma,

Shanghai, China. Antibody was purchased from vendors of the products shown in Table S3, used according to manufacturer's instructions. Insulin Mouse ELISA Kit (Catalog # EMINS) was from ThermoFisher Scientific.

Supplemental Methods

Plasmid construction

Mouse MRPS6, SLC5A3 and ATF5 cDNAs were amplified by polymerase chain reaction (PCR) with primers bearing EcoRI (MRPS6 and ATF5) or NotI (SLC5A3) restriction sites and cloned into pcDNA3.1 vector. The plasmids were then subject to Sanger sequencing to verify the correct insertion direction.

Supplemental Tales

Table S1, siRNAs

Gene	siRNA	Reference
Mm_MRPS6	5'-GUGCUGUGGAGAACAUACUdTdT-3'	This study
	5'-AGUAUGUUCUCCACAGCACdTdT-3'	
Mm_MRPS5	5'-GGACATACAATATTCCATGdTdT-3'	This study
	5'-CAUGGAAUAUUGUAUGUCCdTdT-3'	
Mm_SCL5A3	5'- GACATCCATTATATGTACGdTdT-3'	This study
	5'- CGUACAUAUAAUGGAUGUCdTdT-3'	
Mm_HSPA9	5'- GCUAUGCAGGAUGCAGAAGdTdT-3'	This study
	5'- CUUCUGCAUCCUGCAUAGCdTdT-3'	
Mm_ATF5	5'- GGACCUGCUAAUUGAGGUGdTdT-3'	This study
	5'- CACCUCAAUUAGCAGGUCCdTdT-3'	

Table S2, qPCR primers

Gene	Primer set	Reference
Mm_GAPDH	5'-CAAGGACACTGAGCAAGAGA-3'	[1]
	5'-GCCCCTCCTGTTATTATGGG-3'	

Mm_HSPD1	5'-ACGATCTATTGCCAAGGAGG3'	[1]
	5'-TCAGGGGTTGTCACAGGTTT-3'	
Mm_HSPA9	5'- CTCTGGGAGGCGTCTTTACC	[2]
	5'- CGTTCCCCCTGACACACTTT	
Mm_CLPP	5'- TGCAGTACATCCTGAACCCC-	[2]
	5'- CTCTGCTTGGTGTGTTTGGC	
Mm_LONP1	5'- CGGATGTGTTTCCTCACCTG -3'	[3]
	5'- ACGCCAACATAGGGCTGTG-3'	
Mm_INS1	5'-CTCCCAAAGGGCAAGCAG-3'	[4]
	5'-GTGACCAGCTATAATCAGAGACCA-3'	
Mm_INS2	5'-GGTCTGAAGGTCACCTGCTC-3'	[4]
	5'-GGAGCGTGGCTTCTTCTACA-3'	

Table S3 antibodies

Antibodies	Vendor name	Catalog #
MRPS6	Antibodies-Online	ABIN6144048
SLC5A3	Novus Biologicals	NBP1-02399
Proinsulin	R & D systems	MAB13361
LonP1	Novus Biologicals	NBP1-81734
mtHsp70	ThermoFisher Scientific	MA3-028
Hsp60	ThermoFisher Scientific	MA3-012
ClpP	Novus Biologicals	NBP215919
ATF5	Novus Biologicals	NBP1-51908
Tubulin	Sigma	ZRB1416
Insulin	R & D systems	MAB1417
Glucagon Alexa488	R & D systems	IC1249G
Insulin-APC	R & D systems	IC1417A

Supplemental Figures

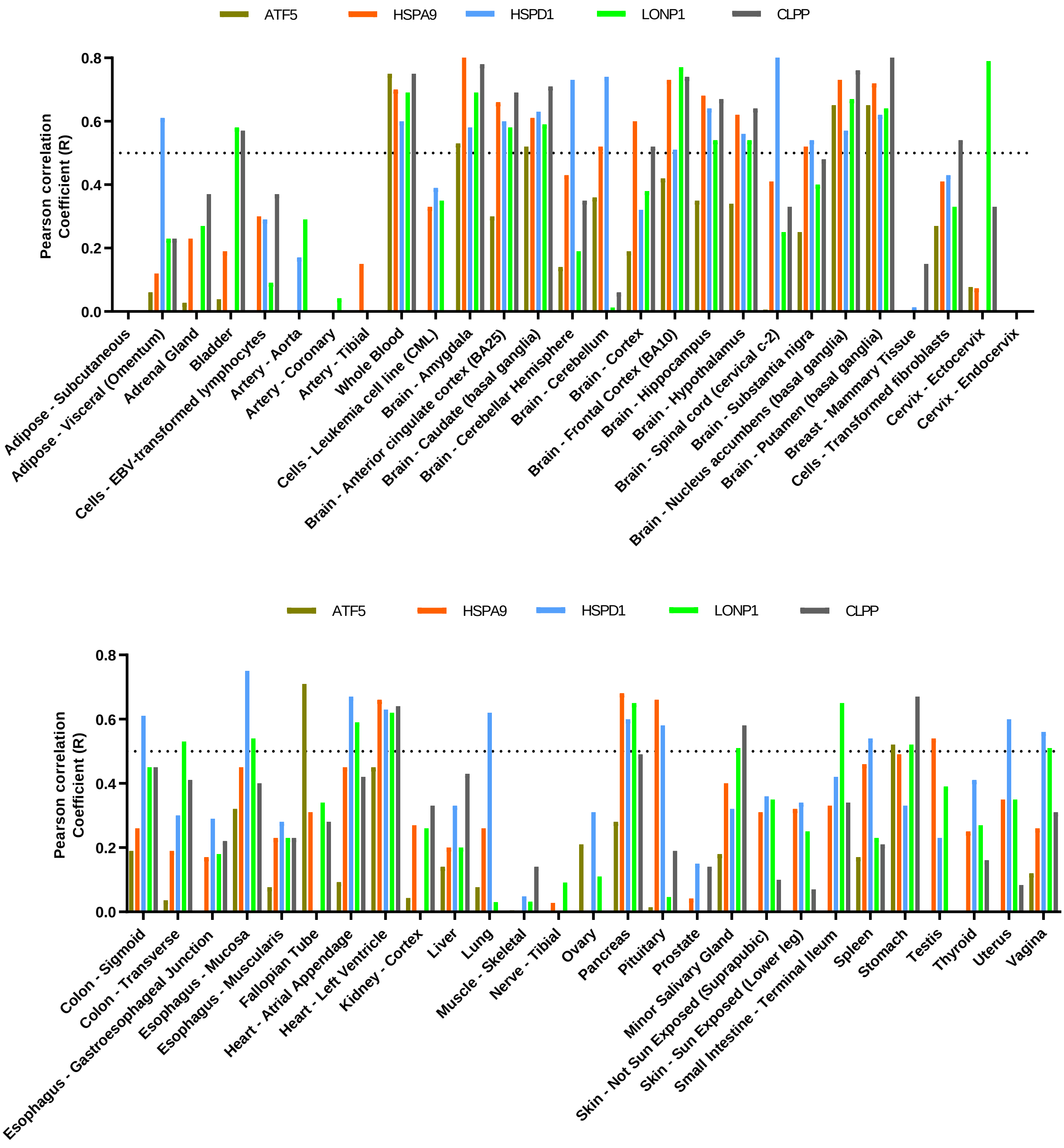

Figure S1. Complete dataset supplementary Figure 2A. Expression correlation of MRPS6 with UPRmt marker genes (*HSP9A*, *HSPD1*, *LONP1* and *CLPP*) across multiple human tissues. Graphs were generated by plotting Pearson correlation efficient generated from GEPIA website

using datasets from human pancreas tissues in the GETx database. R stands for Pearson correlation coefficient indicating the degree of association: “0” denotes no association and “1” denotes perfect positive association.

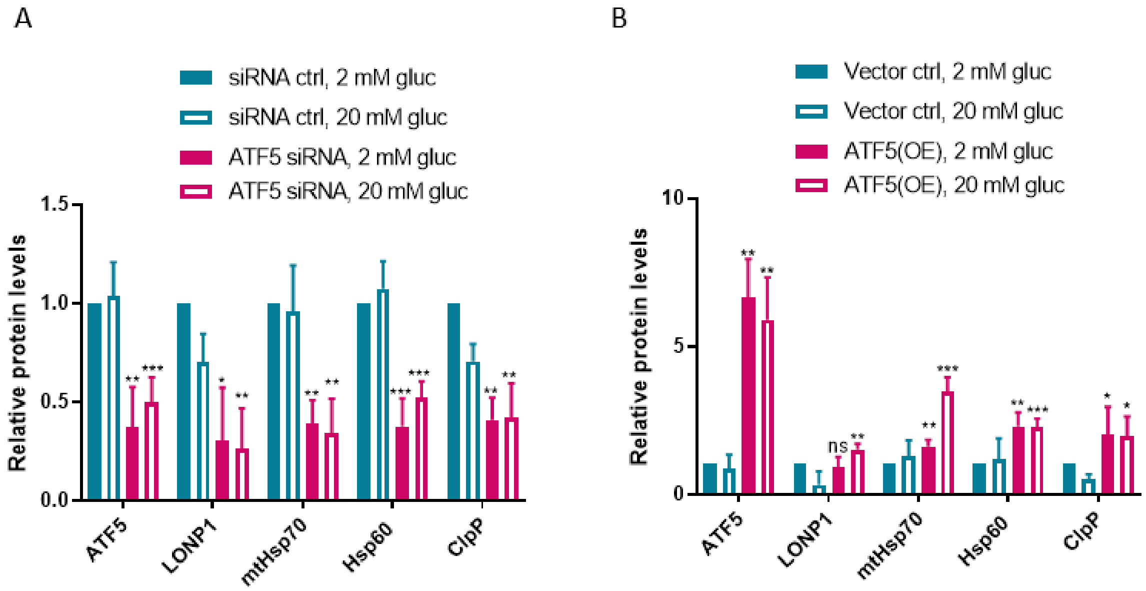

Figure S2. ATF5 knockdown by siRNA and overexpression (OE) effectively inhibits and activates, respectively, UPR^{mt} marker (mtHsp70, Hsp60, LONP1, CLPP) in low (2mM) and high (20 mM) glucose treated β-cells. Representative immunoblots are shown in Figures 4A-B. All experiments were performed >3 biological repeats and error bars show standard deviation (SD) of the mean. P values are based on Student’s t-test: ns denotes not significant, * denotes P<0.05, ** denotes P<0.01, *** denotes P<0.001.

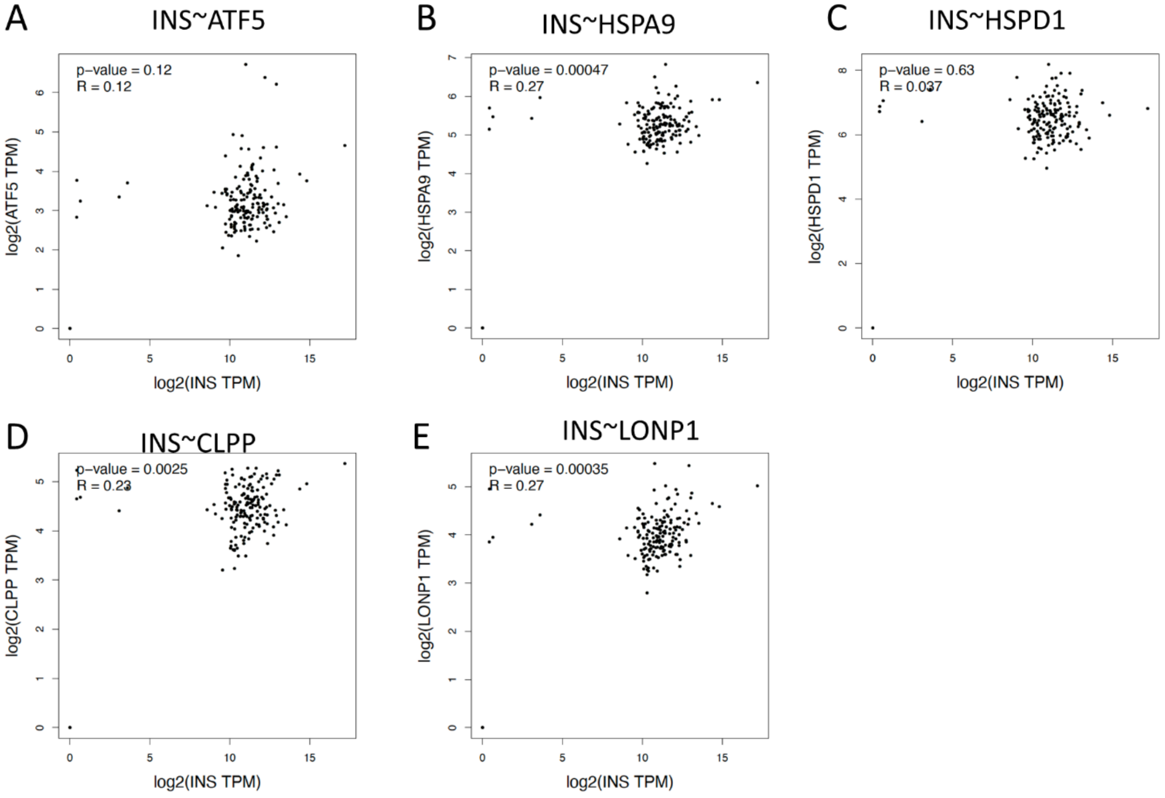

Figure S3. The expression of human insulin gene (*INS*) was not correlated with UPR^{mt} transcriptional factor ATF5 (A) and UPR^{mt} marker genes *HSP9A* (B), *HSPD1* (C), *LONP1* (D), and *CLPP* (E). Graphs were generated by plotting Pearson correlation efficient generated from GEPIA website using datapoints from human pancreas tissues in the GETx database R stands for Pearson correlation coefficient indicating the degree of association: “0” denotes no association and “1” denotes perfect positive association.

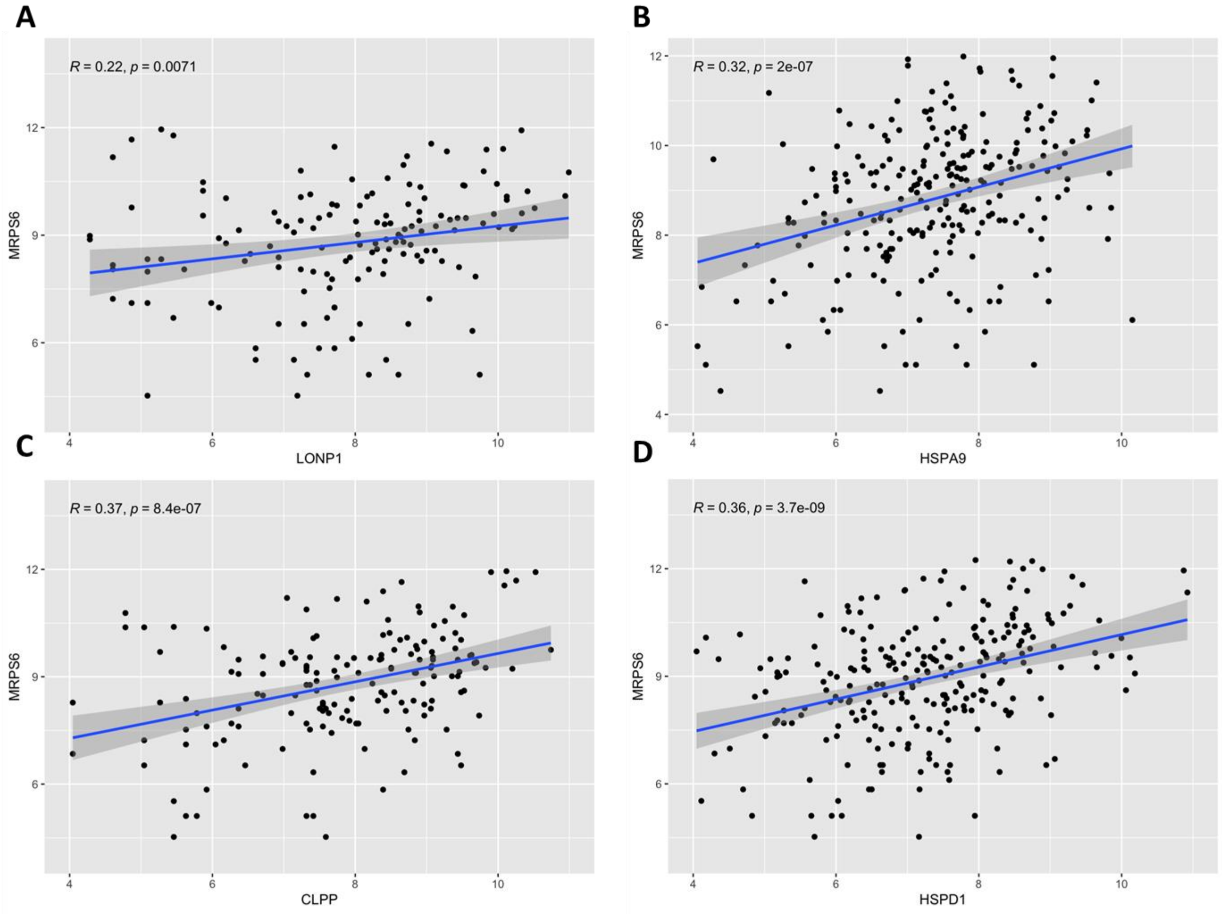

Figure S4. MRPS6 expression was correlated with UPR^{mt} marker genes *LONP1* (A), *HSP9A* (B), *CLPP* (C), and *HSPD1* (D) in human β-cells. GEO dataset GSE124742 are single cell RNAseq samples of human islets of 31 donors. β-cells samples were selected based on the well-established marker INS by log2(CPM) >10. Each data point represents a transcriptome of a single β-cells. Pearson correlation efficient (R) and P values of gene pairs were determined by RStudio. Normalized CPM was log2 transformed and scatter plotted with linear regression by ggplot2 in RStudio.

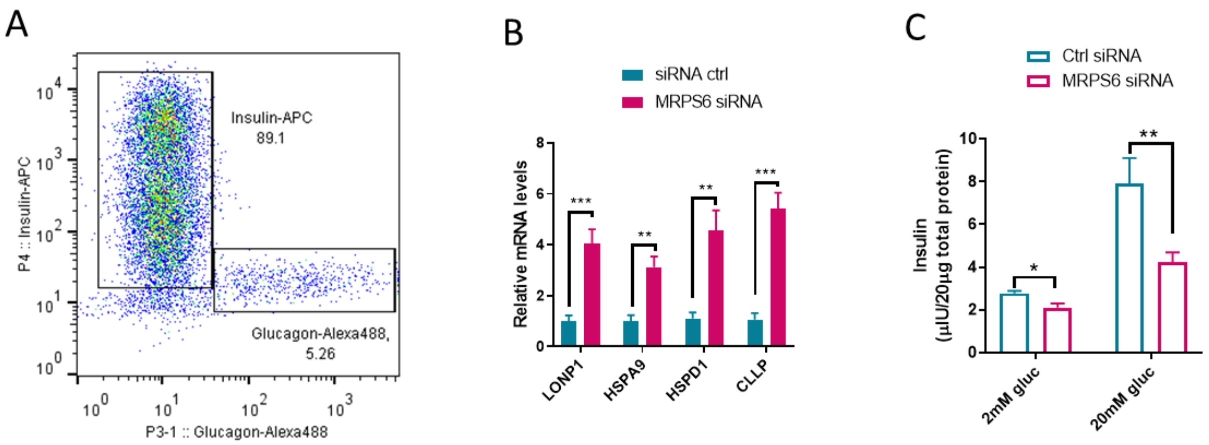

Figure S5. mRPS6 knockdown induced UPR^{mt} and impaired GSIS in mouse primary β-cells. Primary β-cells were isolated by Procell Life Science & Technology from mouse pancreatic tissues. (A) Purity was confirmed by staining cells with an APC-conjugated antibody against insulin (β-cell marker) and an Alexa488 conjugated antibody against glucagon (α-cell marker). (B) Primary β-ells were transient transfected with mRPS6 siRNA for 3 days and RT-qPCR was performed to detect mRNA levels of UPR^{mt} marker genes (LONP1, HSPA9, HSPD1, CLLP). (C) Primary β-ells with and without mRPS6 knockdowns were tested for GSIS by method described in this manuscript.

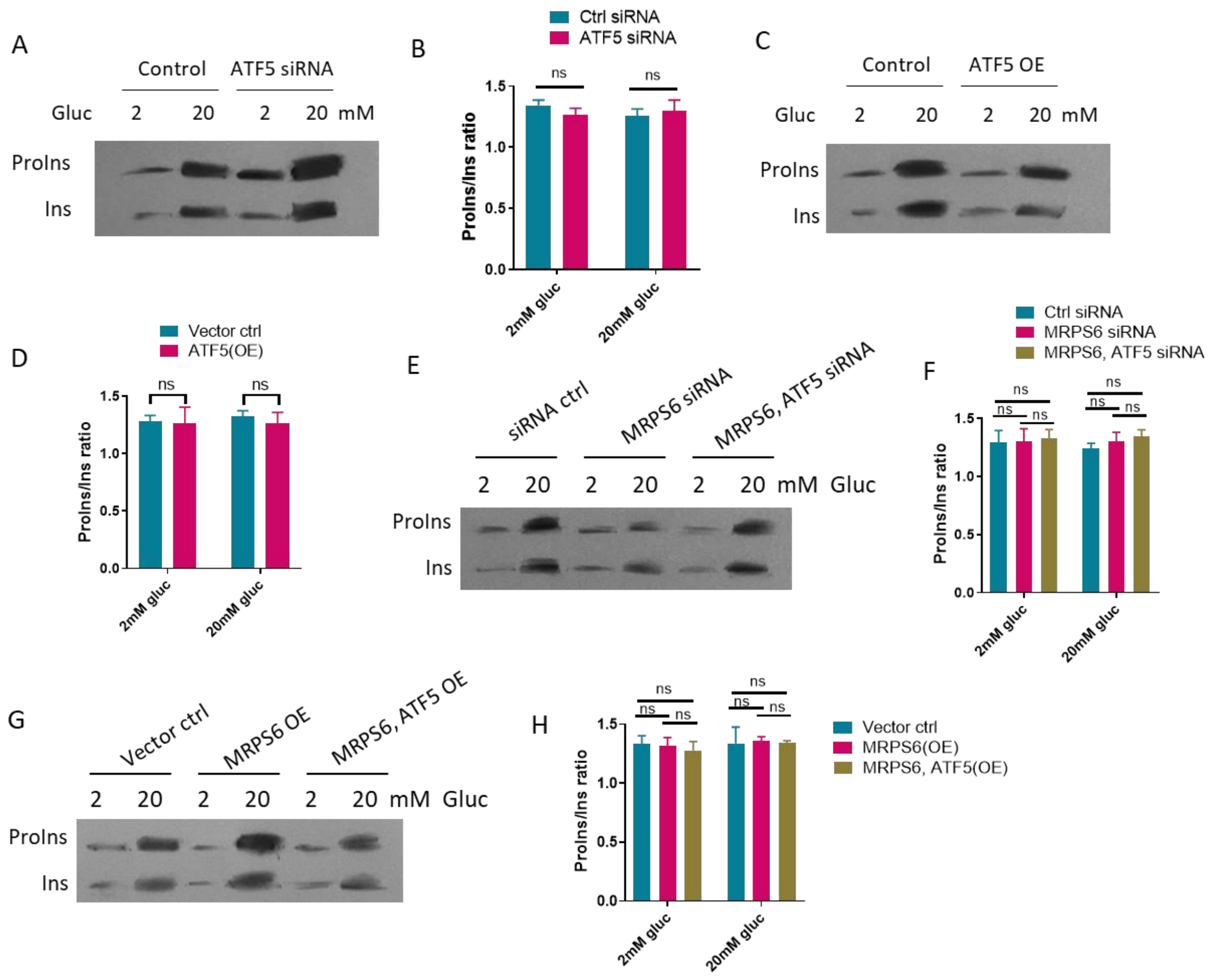

Figure S6. mRPS6 knockdown did not inhibit proinsulin processing. Protein samples from Figure 4 Western blot were analyzed using an antibody recognizing both proinsulin and mature insulin at the same blot. Proinsulin to insulin ratio (ProIns/Ins) were quantified by ImageJ. (A-B) are samples for Figure 4A. (C-D) are samples for Figure 4B. (E-F) are samples for Figure 4G. (G-H) are samples for Figure 4H. Blots show representative blotting data bar graphs show the quantification of 3 repeats. One-way ANOVA with Turkey's multiple comparison test: ns, not significant.

Figure S7. Uncropped immunoblots supplemental to Figure 1B, 1D, 1G, 1J, 3A, 3D, 3G, 4A, 4B, 4G, and 4H. Proteins samples were subject to SDS-PAGE then transferred to PVDF membrane. The membranes were blocked in 5% non-fat milk for 1 hr then probed with primary antibodies in 5% non-fat milk for 1 hr. The membranes were washed with PBST (PBS+0.5% Tween-20) for 5 times, 10 min each time, then incubated with HRP-conjugated secondary antibodies. After extensive washing as above, the membranes were developed by enhanced chemiluminescence (ECL) and pictures were taken. Protein samples were indicated with red on the top of each blot. The sample MRPS5 in 3A were initially labeled with mistakes (handwritings: SCL5A3) then corrected to MRPS5.

Figure 1B: MRPS6

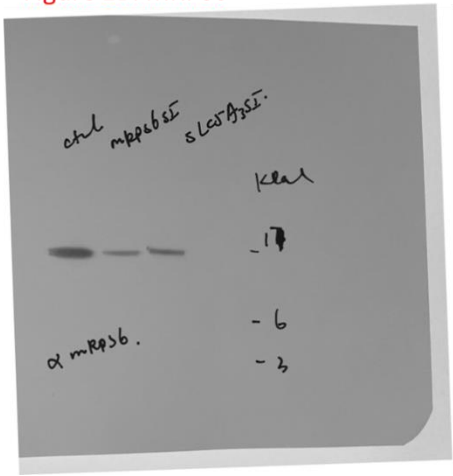

Figure 1B: SLC5A3

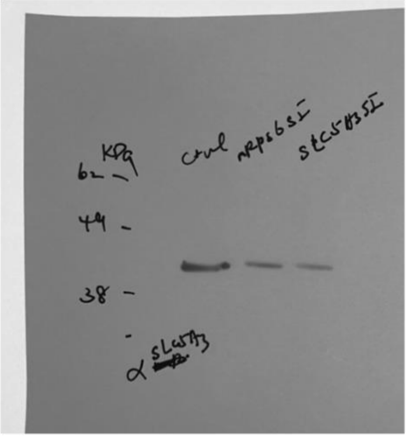

Figure 1B: Tubulin

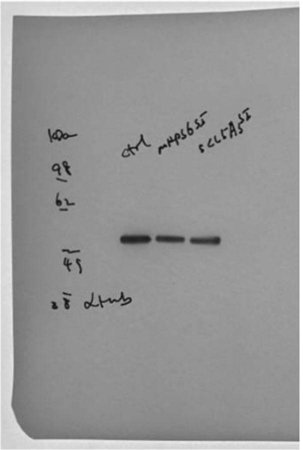

Figure 1D: MRPS6

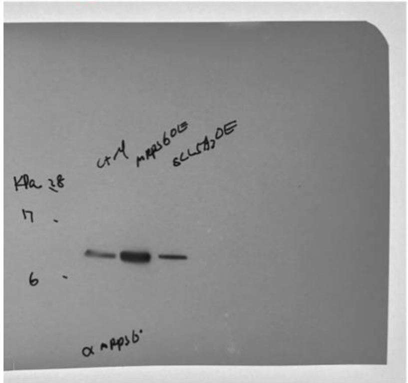

Figure 1D: SLC5A3

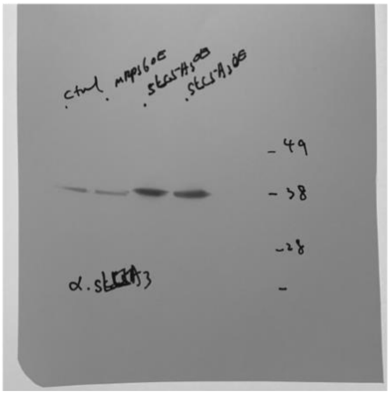

Figure 1D: Tubulin

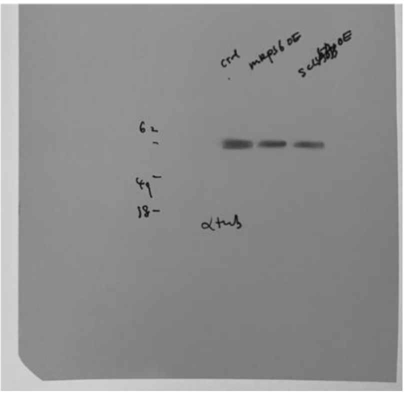

Figure 1G: ProIns

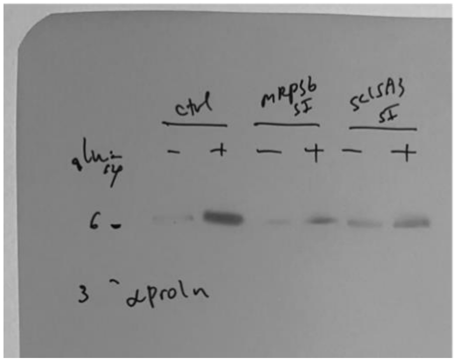

Figure 1G: Tubulin

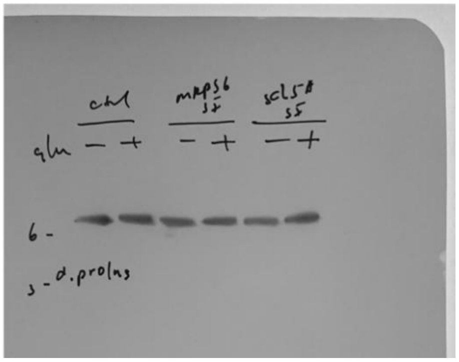

Figure 1J: ProIns

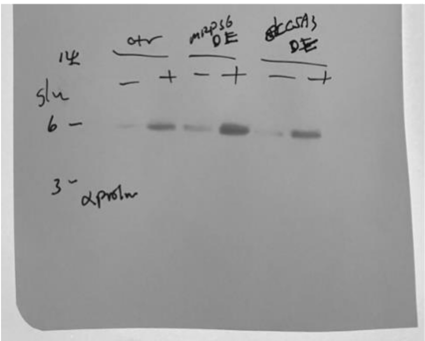

Figure 1J: Tubulin

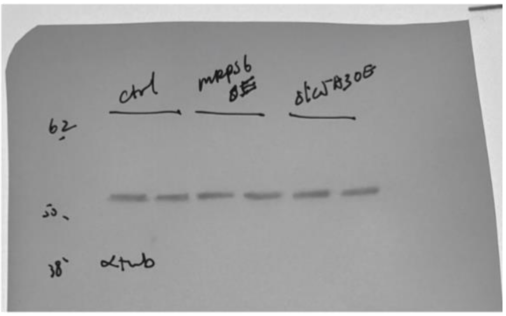

Figure 3A: LonP1

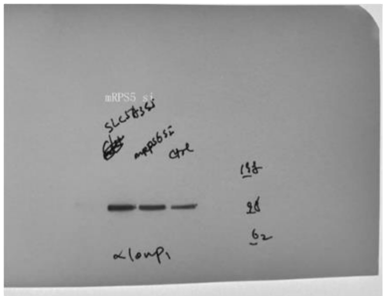

Figure 3A: mtHsp70

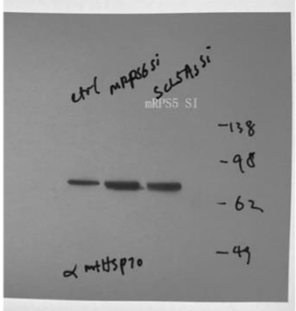

Figure 3A: Hsp60

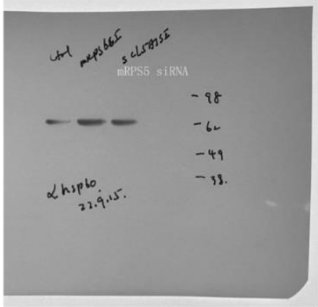

Figure 3A: ClpP1

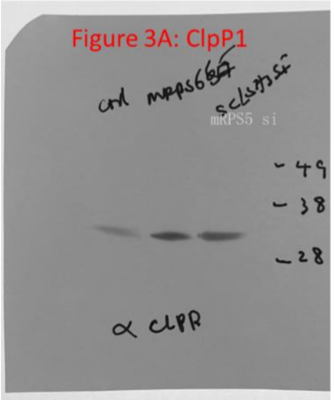

Figure 3A: Tubulin

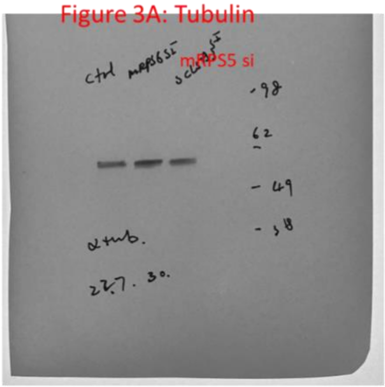

Figure 3D: MRPS6

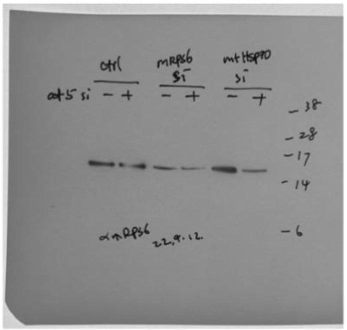

Figure 3D: LonP1

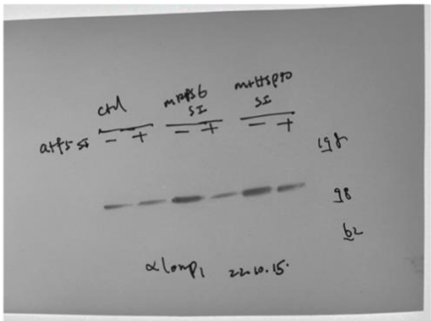

Figure 3D: mtHsp70

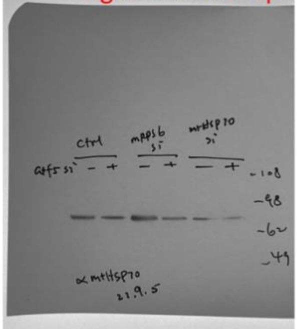

Figure 3D: Hsp60

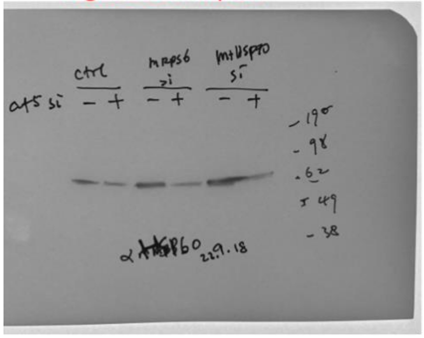

Figure 3D: ClpP

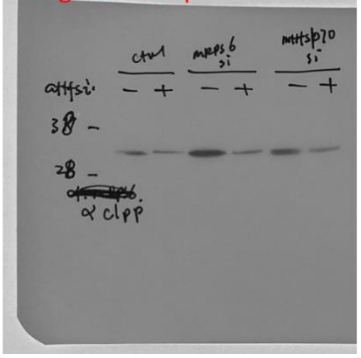

Figure 3D: Tubulin

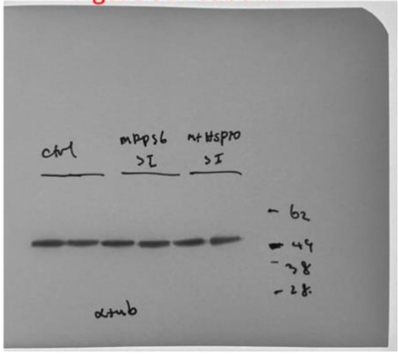

Figure 3G: MRPS6

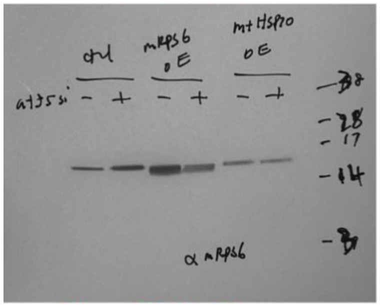

Figure 3G: LonP1

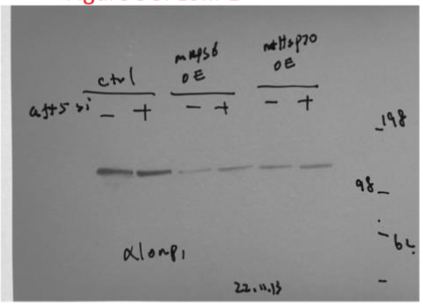

Figure 3G: mtHsp70

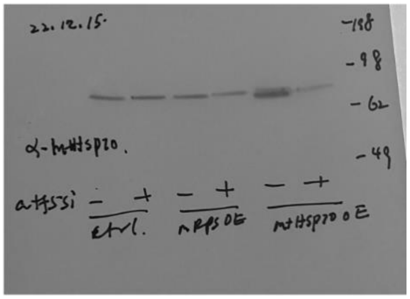

Figure 3G: Hsp60

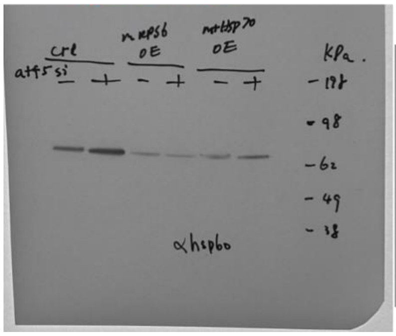

Figure 3G: ClpP1

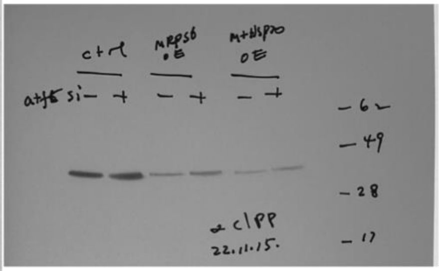

Figure 3G: Tubulin

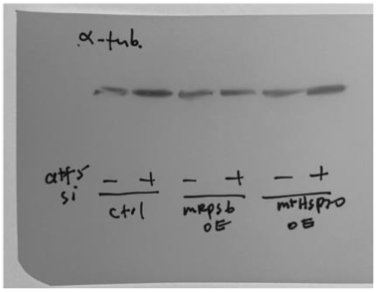

Figure 4A: ATF5

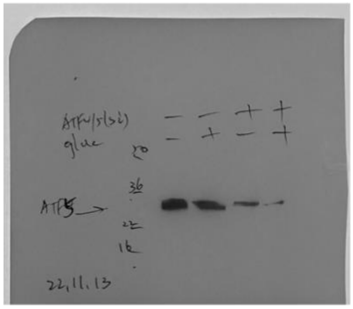

Figure 4A : LonP1

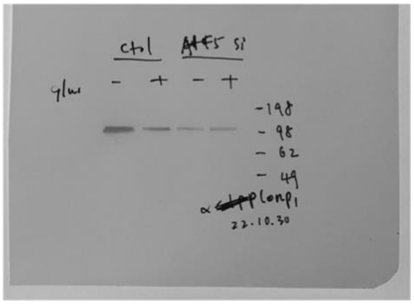

Figure 4A : mtHsp70

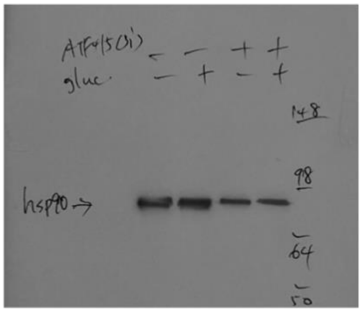

Figure 4A : Hsp60

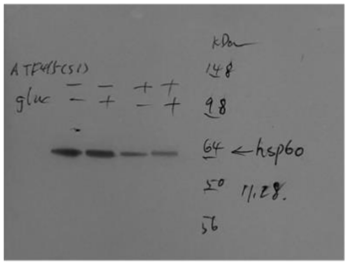

Figure 4A: ClpP1

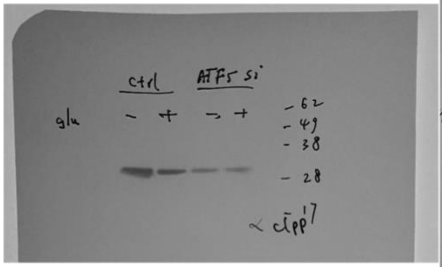

Figure 4A : ProIns

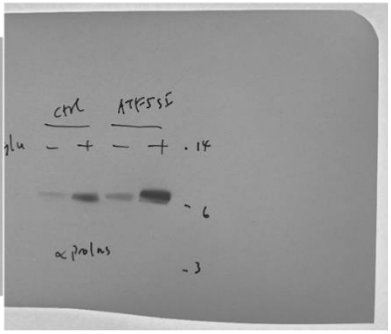

Figure 4A : Tubulin

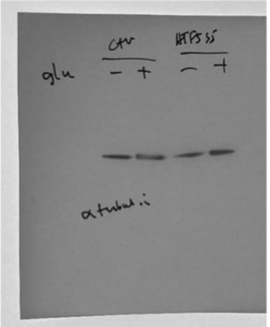

Figure 4B: ATF5

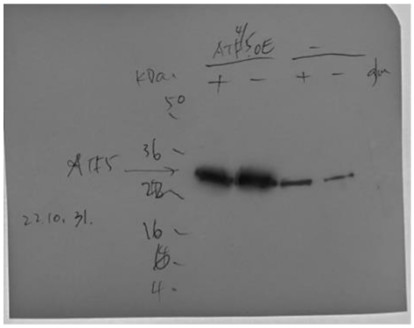

Figure 4B : LonP1

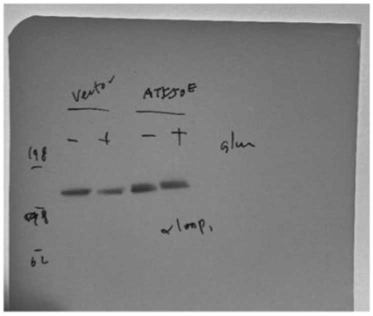

Figure 4B : mtHsp70

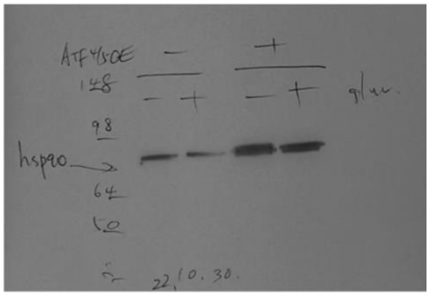

Figure 4B : Hsp60

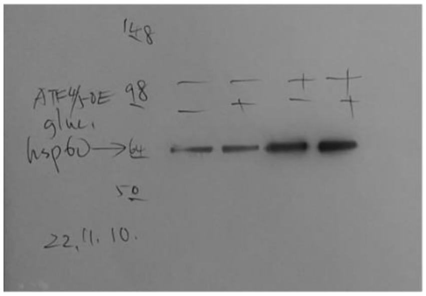

Figure 4B: ClpP1

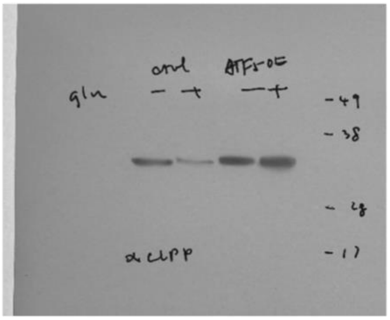

Figure 4B : ProIns

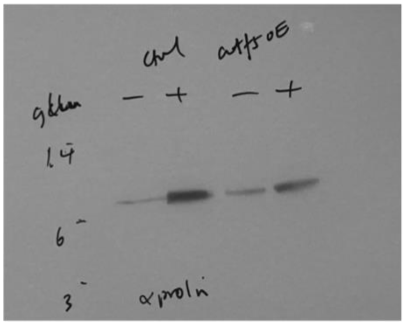

Figure 4B : Tubulin

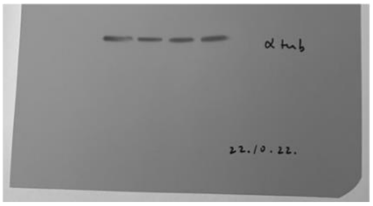

Figure 4G : ProIns

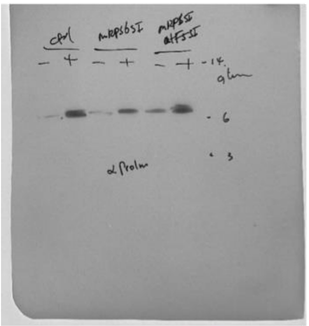

Figure 4H: ProIns

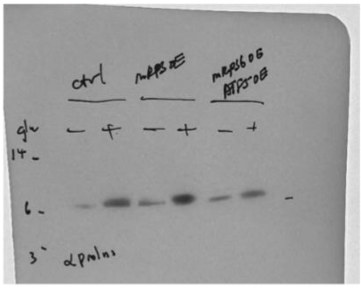

Figure 4G : Tubulin

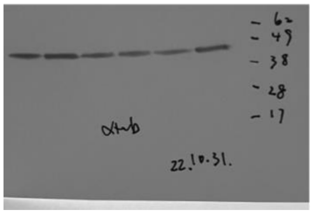

Figure 4H: Tubulin

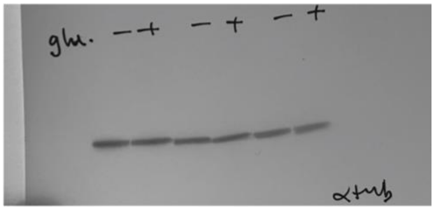

References:

1. Barone, R., et al., *Skeletal muscle Heat shock protein 60 increases after endurance training and induces peroxisome proliferator-activated receptor gamma coactivator 1 alpha1 expression.* Sci Rep, 2016. **6**: p. 19781.
2. Fan, F., et al., *Deletion of heat shock protein 60 in adult mouse cardiomyocytes perturbs mitochondrial protein homeostasis and causes heart failure.* Cell Death Differ, 2020. **27**(2): p. 587-600.
3. Zhao, K., et al., *LONP1-mediated mitochondrial quality control safeguards metabolic shifts in heart development.* Development, 2022. **149**(6).
4. Scavuzzo, M.A., et al., *Pancreatic Cell Fate Determination Relies on Notch Ligand Trafficking by NFIA.* Cell Rep, 2018. **25**(13): p. 3811-3827 e7.
